# Tissue-specific direct targets of *Caenorhabditis elegans *Rb/E2F dictate distinct somatic and germline programs

**DOI:** 10.1186/gb-2013-14-1-r5

**Published:** 2013-01-23

**Authors:** Michelle Kudron, Wei Niu, Zhi Lu, Guilin Wang, Mark Gerstein, Michael Snyder, Valerie Reinke

**Affiliations:** 1 Department of Genetics, Yale University School of Medicine, 333 Cedar Street, New Haven, CT 06520, USA; 2 MOE Key Lab of Bioinformatics and System Biology, Rm 2-111 Biotech Building, School of Life Sciences, Tsinghua University, Beijing, China 100084; 3 Program in Computational Biology and Bioinformatics and Department of Molecular Biophysics and Biochemistry, Yale University, 266 Whitney Avenue, New Haven, CT 06520, USA; 4 Department of Genetics, Stanford University School of Medicine, Mail Stop-5120, Stanford, CA 94305, USA

## Abstract

**Background:**

The tumor suppressor Rb/E2F regulates gene expression to control differentiation in multiple tissues during development, although how it directs tissue-specific gene regulation *in vivo *is poorly understood.

**Results:**

We determined the genome-wide binding profiles for *Caenorhabditis elegans *Rb/E2F-like components in the germline, in the intestine and broadly throughout the soma, and uncovered highly tissue-specific binding patterns and target genes. Chromatin association by LIN-35, the *C. elegans *ortholog of Rb, is impaired in the germline but robust in the soma, a characteristic that might govern differential effects on gene expression in the two cell types. In the intestine, LIN-35 and the heterochromatin protein HPL-2, the ortholog of Hp1, coordinately bind at many sites lacking E2F. Finally, selected direct target genes contribute to the soma-to-germline transformation of *lin-35 *mutants, including *mes-4*, a soma-specific target that promotes H3K36 methylation, and *csr-1*, a germline-specific target that functions in a 22G small RNA pathway.

**Conclusions:**

In sum, identification of tissue-specific binding profiles and effector target genes reveals important insights into the mechanisms by which Rb/E2F controls distinct cell fates *in vivo*.

## Background

The Rb/E2F transcriptional complex is a major regulator of developmental and cellular fates. Underscoring its importance, the pocket protein Rb acts as a key tumor suppressor protein in cancers of diverse tissue origin (reviewed in [1]). Rb acts in large part by regulating the activity of E2F, a heterodimeric sequence-specific DNA binding factor composed of an E2F and DP subunit. In mammals, these factors are members of gene families: there are at least eight E2F-related factors, three DP-related factors, and three pocket proteins. These family members exhibit considerable redundancy and compensation. Moreover, a particular family member can either promote or inhibit tumorigenesis in a cell type-dependent manner (reviewed in [2]). This complexity has greatly hampered a mechanistic understanding of how the Rb/E2F pathway acts *in vivo*. To date, the only genome-wide chromatin immunoprecipitation (ChIP) analyses of mammalian Rb/E2F have been performed in tissue culture, often in transformed cell lines (for example, [3,4]). While valuable, the resulting global DNA binding profiles of Rb and E2F can be correlated only indirectly with tissue-specific phenotypes and ultimately with tumorigenesis.

The nematode *Caenorhabditis elegans *provides an excellent system in which to directly investigate the function of Rb/E2F *in vivo*. Relative to mammals, its Rb/E2F pathway is very streamlined, with only one Rb-like pocket protein (LIN-35), one DP-like protein (DPL-1), and three E2F-related proteins, of which EFL-1 exerts the broadest effects in the animal [5,6]. As in mammals, these factors are broadly expressed and play diverse roles in different tissues. They are part of a gene regulatory pathway known as SynMuv B that mediates differentiation of various somatic tissues, including the vulva, intestine, and pharynx (reviewed in [7]). A recent report used genetic, biochemical and gene expression data to place members of the SynMuv B pathway into three functionally distinct 'complexes', the DRM, heterochromatin, and Mec/Sumo complexes [8]. These three complexes contribute differentially to various SynMuv B phenotypes, potentially by selectively regulating subsets of target genes. LIN-35, EFL-1 and DPL-1 are members of the DRM complex.

A major function of the SynMuv B pathway is to prevent somatic tissues from adopting characteristics of the germline fate, such as ectopic expression of germline genes, enhanced response to RNA interference (RNAi), and increased transgene silencing [9]. This soma-to-germline transformation is also associated with disrupted intestinal function and larval arrest at high temperatures [10]. Intriguingly, certain members of both DRM and heterochromatin complexes, such as *lin-35 *and *hpl-2*, respectively, are required for the high temperature arrest, while other members of the two complexes, such as *efl-1 *and *lin-61*, are not [10], suggesting tissue-specific formation of the SynMuv B complexes

Additionally, components of the SynMuv B pathway act differently in the germline compared to somatic tissues. For instance, in *lin-35 *mutants, germ cells exhibit impaired proliferation but can still undergo gametogenesis and fertilization; as a consequence mutants are fertile but have decreased brood size. By contrast, *efl-1 *and *dpl-1 *mutants display severe defects in oogenesis, ovulation, and fertilization, and are sterile [5,11]. All of these data indicate that LIN-35, EFL-1 and DPL-1 control a fundamental developmental choice between 'immortal' germline and differentiated soma. However, an understanding of the tissue-specific relationships between these proteins and their targets remains unclear. In particular, it is essential to determine whether these proteins directly influence many target genes or a few master regulators to determine these fates. To understand mechanistically how LIN-35, EFL-1 and DPL-1 mediate their diverse effects in different contexts, we have chosen to identify the target sites for these factors in multiple tissue types.

Genome-wide analysis of transcription factor binding in *C. elegans *has so far only been carried out using whole animals as the source material [12-14]. In particular, one recent study identified binding sites for another DRM component, LIN-54, in whole animals with all developmental stages combined [15]. While providing insight into the organismal function of the SynMuv pathway, this and other studies to date have masked cell type-specific binding events for broadly expressed factors with diverse functions, such as Rb/E2F.

To address this limitation, we selectively expressed epitope-tagged LIN-35, EFL-1, and DPL-1 in the germline, intestine, and throughout the soma. We also expressed the SynMuvB heterochromatin complex protein HPL-2 (HP1-like) in the intestine. With these strains, we profiled chromatin interactions genome-wide and identified binding sites for each factor in each tissue that define sets of tissue-specific target genes with distinct properties and functions. Strikingly, most EFL-1/DPL-1 binding sites in the germline exhibit little to no LIN-35 binding, and LIN-35 binding is impaired overall in the germline relative to the soma. Conversely, in the intestine, LIN-35 binding is robust, and a subset of sites co-bound by HPL-2 but not EFL-1/DPL-1 exhibit unique properties. Our data suggest that LIN-35/EFL-1/DPL-1 most likely inhibits the germline fate in somatic tissues by directly acting on a few key targets rather than on many hundreds of individual genes. In sum, these tissue-specific binding profiles lead to insights into tissue-specific properties of Rb/E2F function.

## Results

### Tissue-specific binding profiles for LIN-35, DPL-1, EFL-1 and HPL-2

We generated a series of tissue-specific transgenes containing the *efl-1, dpl-1 *or *lin-35 *genomic locus with a GFP:FLAG epitope tag inserted in frame at the carboxyl terminus of each gene, followed by the native 3' UTR (Figure S1A in Additional file 1). Different regulatory sequences were used to restrict transgene expression in the germline (*pie-1 *regulatory sequences), intestine (*ges-1 *regulatory sequences), or broadly throughout diverse cell types (endogenous *lin-35, efl-1*, or *dpl-1 *regulatory sequences). We also tagged *hpl-2 *and expressed it in the intestine, where it has a demonstrated genetic interaction with *lin-35 *and plays a role in the high temperature larval arrest phenotype [10,16].

We produced integrated transgenic strains expressing each GFP-tagged protein, all of which localized to nuclei in the expected tissue(s) but not elsewhere (Figure S1B in Additional file 1). Several transgenes were tested for rescue of mutant phenotypes (Figure S2A-E in Additional file 1; Supplemental Materials and methods in Additional file 1). For example, endogenous LIN-35, which is expressed in both the soma and the germline, rescued the somatic *lin-35 *mutant phenotype of high-temperature larval arrest [10], as well as the germline phenotype of reduced fertility [11]. By contrast, germline LIN-35 rescued the reduced fertility, but not the somatic larval arrest, demonstrating tissue-specific function. In sum, the rescue experiments are consistent with tissue-specific activity of the transgenic proteins.

We then selected an appropriate developmental stage to characterize DNA binding for each factor in each tissue. The young adult stage is optimal for germline-expressed factors because animals at this stage have a fully developed germline with ongoing oogenesis but few embryos. We selected the larval L1 stage to analyze both endogenous-expressed and intestine-expressed factors, because animals at this stage have primarily somatic tissues (only two quiescent germ cells are present) [17,18], and the soma-to-germline transformation is best characterized at the L1 stage [10,16]. Thus, the data sets corresponding to the endogenous-expressed and intestine-expressed factors will be referred to as 'somatic' and 'intestinal', respectively.

**Figure 1:**
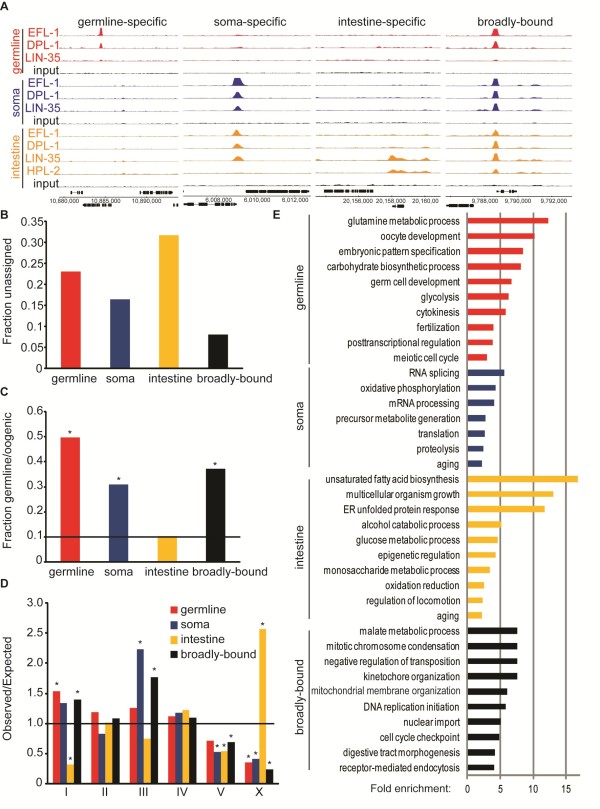
**Genes with tissue-specific binding by LIN-35, EFL-1 and DPL-1 exhibit unique properties**. **(a) **Single-gene examples of the typical binding profile for each tissue-specific dataset. The key for each factor and tissue is to the left of the tracks. One track is shown for each factor in sets corresponding to each tissue, with a control (input) sample for each tissue (black). Red, germline-specific promoter; blue, endogenous promoter; orange, intestine-specific promoter. **(b) **Graph showing the fraction of binding sites for each tissue-specific dataset not readily assignable to at least one nearby coding gene. **(c) **The fraction of candidate gene targets with germline-intrinsic or oogenesis-enriched expression based on [21] (about 0.105 of genes in the genome, marked by black line). Bars marked with an asterisk have significant over-representation (*P *< 1.8 × e-15 or lower; hypergeometric probability test). **(d) **The chromosomal distribution of candidate gene targets for each tissue-specific dataset. Statistically significant deviations from the expected value of 1 (marked with a black line) are indicated by an asterisk (*P *< 1.0e^-05^, Pearson's chi-square). **(e) **Gene Ontology analysis of candidate target genes in each tissue, with Gene Ontology category 'molecular process', and the extent of enrichment indicated by the bar. Up to ten categories, all with more than two-fold enrichment and a *P*-value < 0.05, are shown. Redundant categories were removed manually for each tissue. Full analysis available in Additional file 5.

Biological replicates of synchronized populations of each strain at the selected stage were subjected to ChIP using an anti-GFP antibody, followed by Illumina deep sequencing [12,14]. Several example binding profiles are shown in Figure 1a. Reproducibility between biological replicates was > 90%, except for germline LIN-35 (73%) and intestinal HPL-2 (65%) (Figure S3A in Additional file 1), which showed distinct binding profiles from the other factors. As an additional control, we performed ChIP on wild-type L1 animals using an antibody to the endogenous EFL-1 protein. The binding profile of endogenous EFL-1 was remarkably similar to that of somatic EFL-1 (98% overlap; Figure S3A, B in Additional file 1). This comparison demonstrates that transgenic expression does not result in extensive ectopic binding, validating our approach.

We identified genome-wide binding sites for each factor in each tissue using PeakSeq (q < 0.001) [19]. The number of sites ranged from as few as 688 (germline LIN-35) to as many as 4,055 (somatic LIN-35) (Additional file 2). Consistent with the expectation that LIN-35, EFL-1, and DPL-1 act in a complex, binding sites of these factors exhibit extensive overlap in each tissue (Figure S4 in Additional file 1; Additional file 2), greater than for an unrelated transcription factor such as ALR-1 (data not shown) [14].

### Tissue-specific gene targets have distinct properties and functions

Each factor clearly exhibited tissue-specific binding events (Figure S4 in Additional file 1). We formally defined mutually exclusive sets of tissue-specific binding sites using criteria based on known or expected functions of, and relationships between, factors in each tissue as briefly outlined below (see Supplemental Materials and methods in Additional file 1 for additional rationale for criteria).

Germline-specific sites (415) were bound by both germline EFL-1 and germline DPL-1 but not somatic DPL-1. We did not require binding by germline LIN-35 because it displays weak binding in the germline (see below). Somatic DPL-1 had very strong binding and was used to exclude binding sites not specific to the germline.

Soma-specific sites (282) were bound by somatic EFL-1, somatic DPL-1 and somatic LIN-35, but not germline EFL-1 or intestinal HPL-2. Somatic LIN-35, DPL-1, and EFL-1 showed very coordinated binding; thus, we included binding by all three factors. Exclusion of germline EFL-1 sites eliminates those also bound in the germline, while exclusion of intestinal HPL-2 sites removed many known to be non-specific 'HOT' sites [13]. These sites could be occupied in one or more somatic cell types.

Intestine-specific sites (656) were bound by both intestinal LIN-35 and intestinal HPL-2 but not somatic EFL-1 or intestinal EFL-1. This class was defined by our observation of binding sites with unique characteristics in the intestine that lacked EFL-1 binding but had highly coordinated LIN-35 and HPL-2 binding.

Broadly bound sites (1,419) were bound by germline EFL-1, intestinal DPL-1, and somatic LIN-35. These three factors exhibited the strongest binding of any factor in each tissue and were therefore selected to identify binding sites expressed in both the germline and at least one somatic tissue (the intestine), and possibly other somatic tissues as well.

Examples of each category are displayed in Figure 1a. We independently validated a subset of germline-specific and soma-specific sites by ChIP-quantitative PCR (qPCR), confirming tissue-specific binding for 11 of 12 (Figure S5 in Additional file 1). Binding sites in each category were then associated with candidate target genes whose transcript start sites were either less than 500 bp from the binding site (high confidence targets), or between 500 and 2,000 bp from the binding site (low confidence targets). Sites more than 2,000 bp from the start site of any known gene were left unassigned (Additional file 3) [14]. Most were assigned with high confidence to one or more coding genes; however, the intestine-specific dataset exhibits a relatively high fraction of unassigned binding sites (Figure 1b).

To assess the sensitivity of this tissue-specific approach, we compared the tissue-specific datasets with those identified in whole animal, mixed stage ChIP-chip experiments for LIN-54, another SynMuvB component that is expected to share many binding sites with Rb/E2F components as part of the DRM complex [15]. Strikingly, only 9% of the intestine-specific and 11% of the germline-specific targets were identified in the LIN-54 study, compared to 60% and 41% of the broadly bound and soma-specific targets, respectively (Additional file 4). The tissue-specific profiles identify hundreds of new binding sites, in addition to permitting the assignment of many binding events to a particular cell type.

LIN-35, EFL-1 and DPL-1 are expected to regulate genes with germline expression in both the germline and soma [11,20]. We used published germline expression data [21] to assess the fraction of germline-expressed genes for each set of targets, and found that germline expression is over-represented among the germline-specific, soma-specific, and broadly bound targets, but not the intestine-specific targets (Figure 1c). Enrichment for binding at germline-expressed genes in the soma-specific and broadly bound datasets is consistent with the ability of *lin-35 *to repress the germline fate in somatic tissues [9]. Additionally, the germline-specific, soma-specific, and broadly bound candidate target genes are strikingly under-represented on the X chromosome, whereas intestine-specific targets are substantially enriched on the X chromosome (Figure 1d). This observation is consistent with the fact that relatively few germline-expressed genes are located on the X, and the X chromosome is poorly expressed in most germ cells [22,23]. Thus, LIN-35, EFL-1, and DPL-1 primarily bind near germline-expressed genes, with the exception of the intestine-specific sites.

Despite having the common characteristic of germline expression, the genes in the tissue-specific datasets have strikingly different predicted functions, based on Gene Ontology (GO) categories (Figure 1e; Additional file 5). The germline-specific candidate targets include many whose functions have been implicated in oogenesis, fertilization, and embryonic patterning, consistent with the known germline phenotypes of *efl-1 *and *dpl-1 *mutants [11,24]. For example, multiple genes in this dataset mediate chitin and chondroitin biosynthesis and have been implicated in eggshell formation, including *cpg-2, cpg-3, cpg-4, gna-2, cbd-1, chs-1*, and four C-type lectin genes. Soma-specific candidate targets function in fundamental cellular processes that occur in somatic tissues as well as in the germline, such as splicing, translation, and proteolysis. The broadly bound candidate gene targets tend to function in cell cycle-related processes such as mitosis and replication, similar to the best-studied Rb/E2F targets in mammalian systems [3]. Finally, the intestine-specific set is enriched for genes involved in cellular metabolism, such as fatty acid biosynthesis and glucose metabolism, as well as the unfolded protein response in the endoplasmic reticulum. Cumulatively, these results demonstrate that the sets of tissue-specific binding sites correspond to target genes with fundamentally distinct properties.

### LIN-35 exhibits reduced binding in the germline relative to somatic tissues

**Figure 2:**
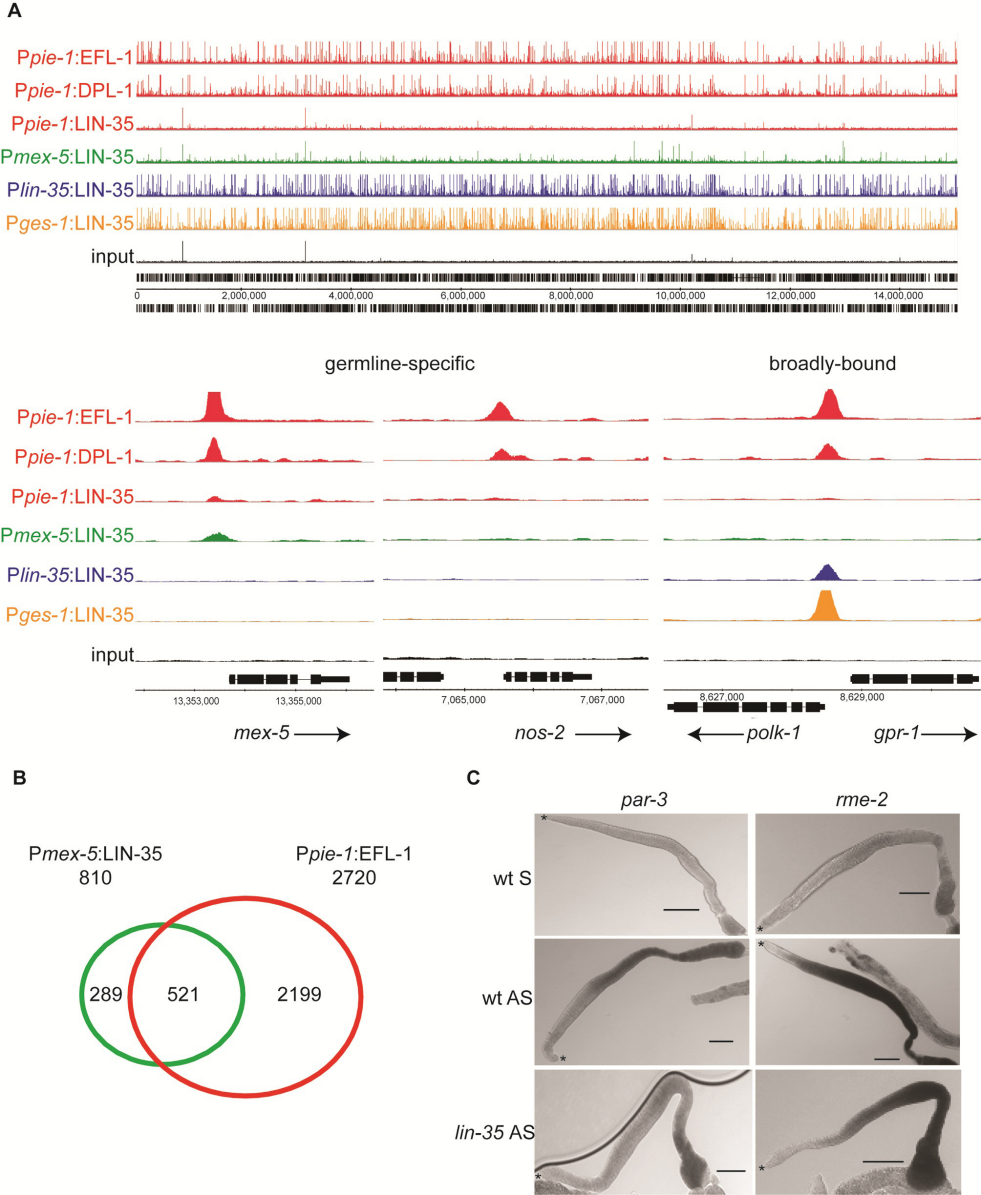
**LIN-35 exhibits reduced binding in the germline**. **(a) **Example of binding across the entirety of chromosome I for each factor in the germline (indicated by the promoter, either P*pie-1*, red, or *Pmex-5*, green), and for LIN-35 in the soma (*Plin-35*, blue) and the intestine (P*ges-1*, orange). Three individual genes are shown below, two of which are germline-specific and show little (left) or no (middle) LIN-35 binding, and one that is broadly bound and shows LIN-35 binding in somatic tissues and not in the germline. **(b) **Venn diagram comparing overlap of binding sites between P*mex-5*:LIN-35:GFP and P*pie-1*:EFL-1:GFP. **(c) ***In situ *hybridization of candidate germline-specific genes (*par-3 *and *rme-2*) in wild-type (wt) and *lin-35 *gonads. AS, antisense probe; S, sense probe. Distal tip marked by an asterisk. Scale bars = 50 μm.

A notable feature of our data was marked reduction of DNA binding by germline LIN-35 compared to somatic tissues (Figure 2a). The relatively few germline LIN-35 binding sites are also bound by germline EFL-1 and/or DPL-1, but are much weaker (median q-value = 6.3e^-08 ^for LIN-35, compared to 6.4e^-23 ^for DPL-1 and 5.2e^-95 ^for EFL-1; Figure S4 in Additional file 1). Poor binding is likely not a technical problem, because germline LIN-35 rescued the *lin-35 *mutant phenotype in the germline (Figure S2 in Additional file 1). Moreover, the epitope tag does not impair LIN-35 binding, as both somatic and intestine LIN-35:GFP bound chromatin proficiently. However, to ensure that the weak binding of germline LIN-35 did not reflect an undetected problem with this particular transgenic line, we utilized the promoter of *mex-5 *to drive LIN-35:GFP expression in the germline; this line also rescued *lin-35 *mutant germline defects (Figures S1 and S2 in Additional file 1). ChIP-seq analysis demonstrated that *mex-5*-driven LIN-35:GFP exhibits the same limited binding capability as *pie-1*-driven LIN-35:GFP (Figure 2a). Moreover, P*mex-5*:LIN-35:GFP binding sites overlap extensively with binding by germline EFL-1 (Figure 2b), similar to P*pie-1*:LIN-35:GFP (Figure S3A in Additional file 1). This result confirms that the binding capability of LIN-35 is impaired specifically in the germline.

Reduced LIN-35 binding in the germline might occur because LIN-35 has a significantly reduced role in complexes in which EFL-1 and DPL-1 activate gene expression, consistent with the canonical model for Rb/E2F function in which the dissociation of a pocket protein switches E2F from repressor to activator [25]. Alternatively, LIN-35 binding might be restricted to a subset of germ cells. We speculated that LIN-35 might act specifically in the mitotic progenitor cells of the germline to prevent premature activation of EFL-1/DPL-1-regulated genes, which are poorly expressed in progenitor cells and strongly upregulated by EFL-1/DPL-1 as germ cells initiate meiosis and gametogenesis [11]. Consistent with this possibility, the EFL-1/DPL-1 target gene *lip-1 *exhibits increased mRNA and protein levels in the progenitor cells of *lin-35 *mutants [26].

To distinguish between these two possibilities, we performed *in situ *hybridization of wild-type and *lin-35 *mutant gonads to examine the spatial expression pattern of four target genes (*par-3, egg-1, rme-2*, and *chs-1*) bound in the germline by LIN-35, EFL-1 and DPL-1 (Figure 2c; data not shown). However, none exhibited expanded expression into the progenitor cell population of *lin-35 *mutants. Instead, overall expression appeared mildly reduced in the proximal gonad in the mutant compared to wild type. This result is consistent with LIN-35 having a minimal role in complexes in which EFL-1/DPL-1 is functioning as an activator, rather than acting specifically in progenitor germ cells. By contrast, in somatic tissues LIN-35 binding is extensive and the complex primarily inhibits gene expression. Thus, tissue-specific regulation of the association of LIN-35 with EFL-1/DPL-1 might be a key factor determining whether the complex activates or represses gene expression.

### Tissue-specific target genes are differentially regulated in *lin-35, efl-1 *and *dpl-1 *mutants

**Figure 3:**
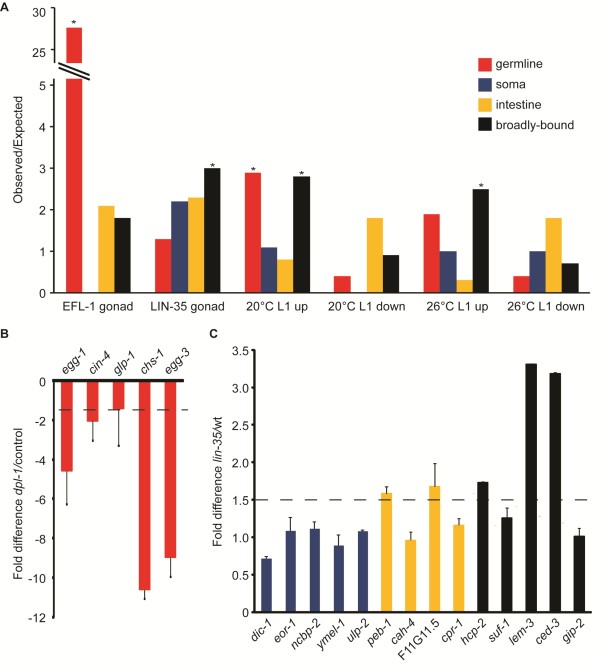
**Tissue-specific target genes in *lin-35, efl-1*, and *dpl-1 *mutants are differentially regulated**. **(a) **Over-representation of tissue-specific candidate gene targets in different published microarray expression datasets. The expression datasets on the x-axis labeled E2F gonad and LIN gonad are from [11], while the 20°C data sets are from [20], and the 26°C data sets are from [10]. Statistically significant deviations are indicated by asterisks (*P *< 1.0e-05, hypergeometric probability test). **(b) **qRT-PCR results for selected germline-specific candidate target genes that were not considered regulated in the gonad microarray datasets. Fold difference of expression was compared between control (*unc-4*) and mutant (*unc-4 dpl-1*) RNA from dissected gonads, and normalized to hexokinase expression. Error bars indicate technical replicates. The dashed line indicates 1.5-fold difference. **(c) **qRT-PCR results for selected soma-specific (blue), intestine-specific (orange), and broadly bound (black) candidate target genes that did not show any regulation in the 20°C or 26°C L1 microarray datasets. The fold difference of expression of each gene was compared between wild-type (N2) and *lin-35(n745) *mutant L1s raised at 26°C, and normalized to actin (*act-3*) expression. Error bars indicate technical replicates. The dashed line indicates 1.5-fold difference.

To investigate how tissue-specific target genes are regulated by EFL-1, DPL-1, and LIN-35, we compared the germline-specific, soma-specific, intestine-specific, and broadly bound targets to data from three published microarray studies on *efl-1, dpl-1*, and/or *lin-35 *mutants, in the same stages and/or tissues (Figure 3a; Additional file 6). One study analyzed gene expression in dissected gonads, identifying 74 genes with down-regulated expression in both *efl-1 *and *dpl-1 *mutant gonads and 88 genes with up-regulated expression in *lin-35 *mutant gonads [11]. Two other studies identified up- and down-regulated genes in the soma of *lin-35 *mutant L1 larvae, either at 20°C [20], or at 26°C [10]. These latter two studies have significant overlap (55% of up-regulated genes from the smaller 20°C gene list; *P *< 8.0e^-224^, hypergeometric probability test).

Comparison of the germline-specific target genes with the genes regulated in dissected gonads of *efl-1, dpl-1 *and *lin-35 *mutants showed that 36 of the 74 down-regulated genes were bound by germline EFL-1 and DPL-1 (*P *< 1.03e^-43^), consistent with EFL-1 and DPL-1 acting directly to promote gene expression in the germline. Only two germline-specific target genes (*unc-101 *and *hsf-2*) were differentially expressed in *lin-35 *mutant gonads, indicating that LIN-35 is not required for the correct expression levels of most targets, and consistent with the limited binding by LIN-35 in the germline. The broadly bound candidate gene targets were over-represented among somatic L1 *lin-35 *up-regulated genes (20°C, *P *< 2.81e^-19^; and 26°C, *P *< 1.82e^-25^), consistent with a subset of these genes being bound and down-regulated by LIN-35 in various somatic tissues. Surprisingly, the soma-specific and intestine-specific targets did not overlap significantly with L1 *lin-35 *regulated genes at 20°C or 26°C.

Indeed, the majority of tissue-specific candidate gene targets from any group were not significantly regulated in the microarray analyses. Transcription factor binding and target gene regulation typically show a poor correlation, which could be due partly to incorrect target assignment or shortcomings with the microarray analysis (reviewed in [27]). We therefore tested whether 'unregulated' direct gene targets were in fact regulated by EFL-1/DPL-1 and/or LIN-35, using qRT-PCR for several candidates in each category. Of five germline-specific targets tested, all five had decreased expression in *dpl-1 *mutant gonads relative to controls (Figure 3b), suggesting that most germline-specific candidate target genes require DPL-1, and presumably EFL-1 as well, for expression in the gonad and that they were missed in the microarray analysis. Additionally, we examined expression of targets in the soma-specific, intestine-specific, and broadly bound sets in *lin-35 *mutant L1 larvae raised at 26°C. Three broadly bound and two intestine-specific genes showed > 1.5-fold increased expression in *lin-35 *mutants relative to wild type (Figure 3c), but the rest exhibited little to no change in expression. This result suggests that LIN-35/EFL-1/DPL-1 inhibits expression of only a subset of the candidate gene targets in these categories. We conclude that a binding event is much more likely to directly affect expression levels of candidate target genes in the germline than in the soma.

Finally, a recent study monitored the expression of several candidate SynMuvB target genes in the soma of young adults lacking a germline, in which various DRM and heterochromatin SynMuvB complexes were inactivated by mutation or RNAi [8]. This analysis defined four DRM-specific targets (*spn-4, mut-2, rde-4*, and *drh-3*), two heterochromatin-complex-specific targets (*wago-1 *and *wago-10*), and seven 'common' targets regulated by both complexes. We therefore examined whether these genes were tissue-specific direct targets of EFL-1, DPL-1, and LIN-35. *wago-1 *and *wago-10 *are germline-specific targets, while *spn-4 *and *mut-2 *are broadly bound, *drh-3 *is not bound, and *rde-4 *exhibits a complex binding pattern in somatic tissues that was not classified. Two of the common targets (*pgl-3 *and *wago-9*) are germline-specific and five were not significantly bound. Thus, we did not find a strict correlation between binding profile and regulation by the different SynMuvB complexes, although notably both heterochromatin complex-specific genes were in the germline-specific category, while none of the DRM-specific genes were.

### *mes-4 *is a direct target of LIN-35/EFL-1/DPL-1 in the soma

The tissue-specific binding profiles permit identification of key targets that might contribute to the adoption of germline-characteristics in the somatic tissues of *lin-35 *mutants. As described above, we found little overlap between *lin-35 *mis-regulated genes and those bound by LIN-35, EFL-1, or DPL-1 in the soma (Figure 3A in Additional file 6), suggesting that LIN-35-mediated repression of germline genes in the soma at this stage is largely indirect.

**Figure 4:**
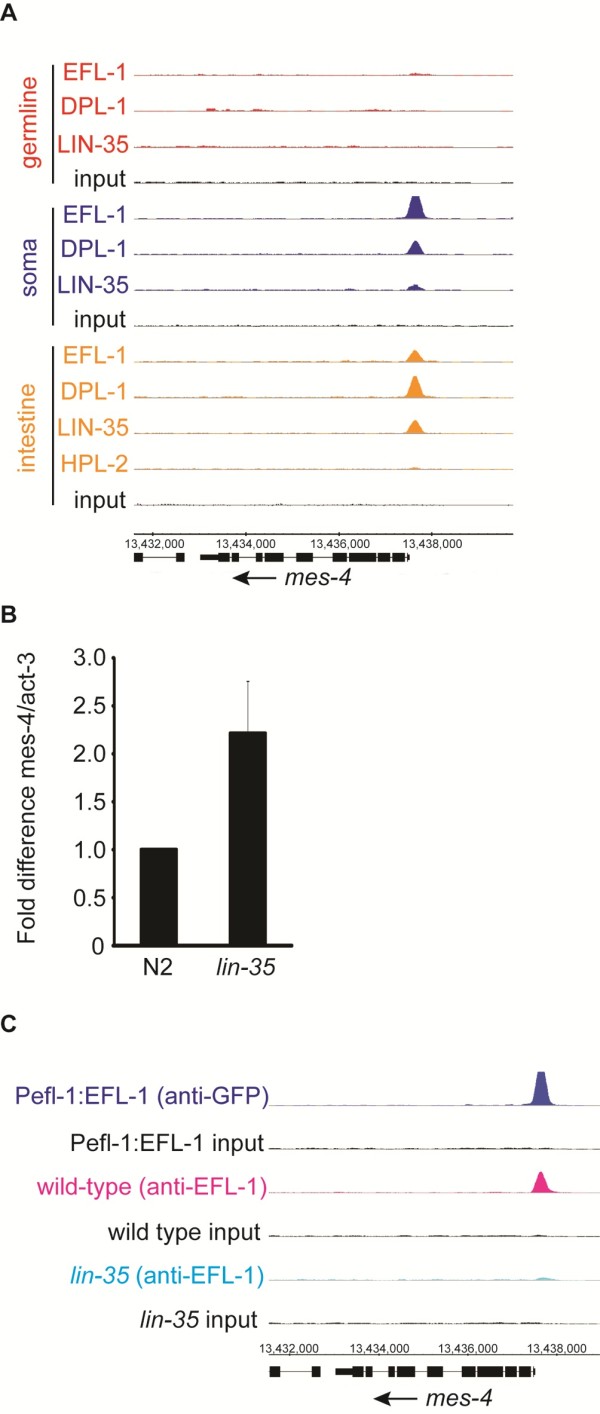
***mes-4 *is a direct target of LIN-35/EFL-1/DPL-1 in somatic tissues**. **(a) **Binding profile of LIN-35, EFL-1, DPL-1 and HPL-2 in multiple tissues at the *mes-4 *locus. **(b) **qRT-PCR verification of up-regulation of *mes-4 *transcript levels in *lin-35 *mutants relative to wild type. The error bar indicates standard error of biological replicates. **(c) **Binding profiles of EFL-1 at the *mes-4 *locus in the soma using the GFP antibody (blue) and in wild-type (magenta) and *lin-35 *(cyan) using the EFL-1 antibody. An input control for each is shown below in black.

Strikingly, one of the few genes both bound by LIN-35 specifically in the soma, and differentially regulated in *lin-35 *mutants, is *mes-4 *(Figure 4a). We confirmed *mes-4 *transcript induction in *lin-35 *mutant L1s relative to wild type by qRT-PCR (Figure 4b), consistent with previous microarray experiments [20]. Moreover, in *lin-35 *mutants, EFL-1 binding at the *mes-4 *promoter is vastly reduced, whereas most other direct target genes still retain EFL-1 binding, suggesting that regulation of *mes-4 *is specifically disrupted in *lin-35 *mutants (Figure 4c; Figure S6A in Additional file 1).

*mes-4 *is especially intriguing as a direct target because its activity is essential for the germline-to-soma transformation: *mes-4*; *lin-35 *mutants exhibit a significantly reduced larval arrest and reduced levels of somatic germ granules relative to *lin-35 *mutants [9,10]. It also has the ability to suppress multiple other *lin-35 *related phenotypes [28]. MES-4 encodes an H3K36 histone methyltransferase that acts primarily in the bodies of genes expressed in the germline, presumably so that they can be re-expressed in germ cells in the next generation [29]. These observations have led to the working model that LIN-35 antagonizes the pro-germline influence of MES-4. Our data suggest that LIN-35 does so, at least in part, by binding directly to the *mes-4 *gene in the soma, to reduce *mes-4 *expression and prevent it from targeting germline-expressed genes inappropriately. In this manner, LIN-35 might prevent activation of an extensive germline gene expression program in the soma without directly binding each regulated gene.

### CSR-1 is required for the soma-to-germline transformation of *lin-35 *mutants

**Figure 5:**
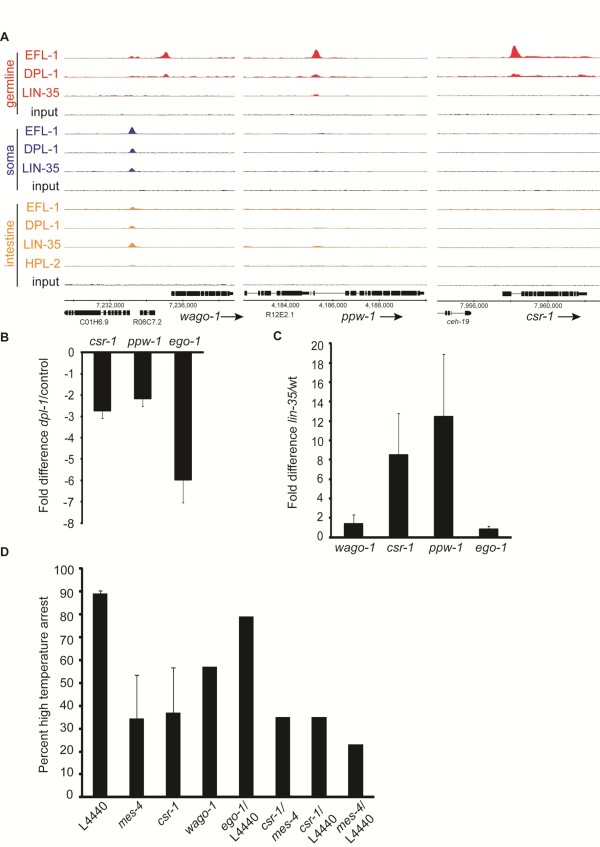
**22G small RNA pathways mediate the *lin-35 *larval arrest phenotype**. **(a) **Binding profiles at loci encoding candidate small RNA pathway regulatory proteins. **(b) **qRT-PCR analysis of transcript levels of candidate small RNA pathway regulators in the dissected gonads of wild-type and *dpl-1 *mutants. Error bars indicate technical replicates. **(c) **qRT-PCR analysis of transcript levels of candidate small RNA pathway regulators in L1 larvae of wild-type and *lin-35 *mutants. Error bars indicate biological replicates. **(d) **Assay of the high temperature larval arrest phenotype of *lin-35 *mutants at 26°C upon RNAi of candidate small RNA regulators (listed on the x-axis) and empty vector control (L4440).

The soma of *lin-35 *mutants also exhibits the germline characteristic of enhanced RNAi sensitivity [9]. One hypothesis is that various proteins involved in RNAi-based pathways in the germline are mis-expressed in the soma of *lin-35 *mutants [9,30]. Examination of the binding profiles of RNAi pathway genes showed that several are bound specifically in the germline, including genes encoding the Argonaute family proteins, *csr-1, ppw-1, wago-1, wago-2*, and *wago-10*, as well as the RNA-dependent RNA polymerase *ego-1 *(Figure 5a; Figure S6B in Additional file 1). To test the effect of binding on their expression in the germline, we performed qRT-PCR on dissected gonads from wild-type and *dpl-1 *mutant animals (Figure 5b). Expression decreased in *dpl-1 *relative to wild-type, indicating that DPL-1, and presumably EFL-1 as well, contribute to germline expression of these genes. In the soma of *lin-35 *mutants, a subset of these genes, primarily *csr-1 *and *ppw-1*, are upregulated (Figure 5c), despite an absence of binding by LIN-35. We suspect that this regulation is stage specific, as Wu *et al. *[8] did not detect consistent regulation of *csr-1 *in young adults. The increased expression of these germline RNAi-related genes in the larval soma could be a consequence of elevated MES-4 activity.

CSR-1 affects chromatin status [31] and chromosome segregation [32], suggesting that its upregulation in the soma might have a significant effect on the soma-to-germline transformation. We therefore tested whether loss of any of these small RNA regulatory genes could rescue the larval arrest phenotype of *lin-35 *mutants at 26°C. We found that loss of *csr-1*, and to a lesser extent *wago-1*, rescued the high temperature arrest of *lin-35 *mutants (Figure 5d). Because CSR-1 binds to small RNAs (22G-RNAs) that are antisense to germline genes, we speculated that these small RNAs might be important for recruiting MES-4 to germline genes to promote their expression, or conversely, that MES-4 activity is necessary for CSR-1 to be appropriately targeted to germline genes. If either of these possibilities is true, then the genes corresponding to CSR-1-bound 22G RNAs should overlap significantly with the genes regulated by MES-4. We therefore compared genes targeted by CSR-1 22G RNAs [32] with MES-4 target genes [29], and found that 76% of the MES-4 targets have CSR-1-associated 22G RNAs (Additional file 7). Moreover, RNAi of *mes-4 *and *csr-1 *together does not further suppress the *lin-35 *larval arrest phenotype compared to RNAi of either gene alone, suggesting that they might act in the same pathway (Figure 5d). Consistent with this possibility, *wago-1(RNAi) *was less effective than *csr-1(RNAi) *at suppressing the *lin-35 *larval arrest, and the overlap between the top 100 genes targeted by WAGO-1 22G RNAs [33] and MES-4 targets was only 10%. We conclude that CSR-1 contributes to the soma-to-germline transformation of *lin-35 *mutants, at least in the intestine.

### LIN-35 and HPL-2 exhibit common specialized binding patterns in intestinal chromatin

**Figure 6:**
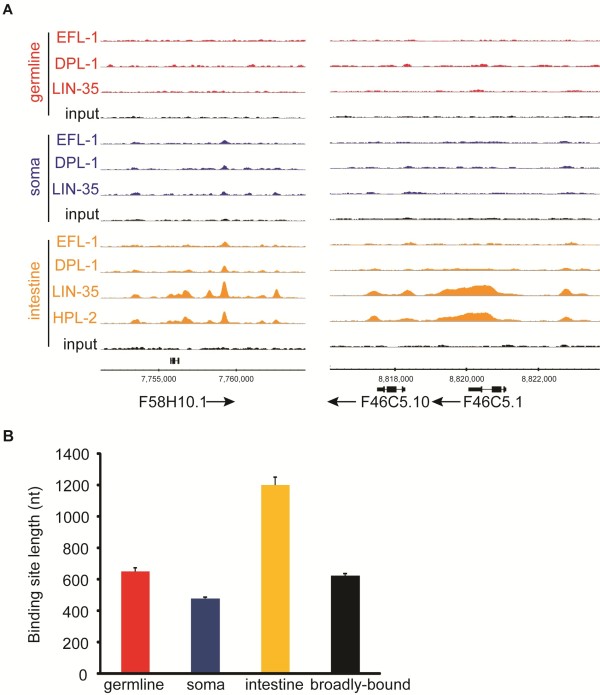
**HPL-2 requires LIN-35 for recruitment to intestine-specific binding sites**. **(a) **Single-gene examples of the typical binding profiles for intestine-specific targets. **(b) **Binding site width for each tissue-specific candidate target set. Error bars represent standard error.

The intestine is the key tissue for mediating the high-temperature larval arrest phenotype of *lin-35 *mutants [10]. The distinction between the DRM and heterochromatin complexes is not consistent in this tissue: for each complex, certain components are involved in the larval arrest (*lin-35 *and *hpl-2*) while others are not (*efl-1 *and *lin-61*) [8,10]. We found that LIN-35 and HPL-2 exhibit a unique type of binding behavior in the intestine that could explain this discrepancy and provide a possible mechanism for the larval arrest phenotype. The subset of binding events we called 'intestine-specific' are bound primarily by LIN-35 and HPL-2, and sometimes exhibit weak binding by DPL-1 but essentially no binding by EFL-1. Thus, these sites have minimal, if any, input by E2F (Figure 6a). As described previously, these intestine-specific binding sites exhibit several other distinctive features, including a high proportion of sites that could not be assigned to specific gene targets, a paucity of target genes with germline expression, and enrichment on the X chromosome (Figure 1b-d). Additionally, fewer genes in the intestine-specific set are associated with E2F consensus motifs (Figure S7 in Additional file 1; Additional file 8). Notably, intestine-specific binding sites cover almost twice as many nucleotides compared to other tissue-specific binding sites (Figure 6b), and are often found in gene bodies or intergenic regions instead of immediate upstream regulatory regions (Figures 1a and 6a).

These observations indicate that LIN-35 is likely recruited to these sites through a novel, intestine-specific mechanism that includes HPL-2. Indeed, we find that HPL-2 binding is diminished in the absence of LIN-35 binding in the intestine (data not shown). Thus, even though HPL-2 might regulate a different set of genes from the DRM complex in other tissues, in the intestine it acts only at a subset of LIN-35 binding events. Possibly, LIN-35 has multiple regulatory functions in the intestine, including a tissue-specific interaction with HPL-2 (either separately or as part of the heterochromatin complex) that is independent of EFL-1, as well as a more canonical, perhaps less tissue-specific, function in the DRM complex.

## Discussion

The Retinoblastoma (Rb) tumor suppressor pathway is inactivated in tumors of diverse tissue origins at a very high frequency. Although intensively studied, the actual mechanisms by which the Rb pathway directs proliferation and differentiation within the tissue-specific restrictions imposed *in vivo *are poorly understood. Here, we address this limitation by developing a system in *C. elegans *to globally identify the tissue-specific chromatin interactions of the core members of this pathway, LIN-35/Rb, EFL-1/E2F, and DPL-1/DP. A key advantage of this approach is that we were able to compare binding profiles between tissues to separate broadly bound sites from those present in individual tissues, thus focusing on the most biologically relevant binding events. This analysis revealed distinct sets of binding sites, with different candidate gene targets and modes of regulation in specific tissues. In a whole animal analysis, the sheer number of broadly bound sites relative to tissue-specific sites would have obscured the distinct functions of the Rb/E2F complex in the different tissues. Many of the broadly bound sites correspond to gene targets related to the best known mammalian E2F targets, such as cell cycle genes. Thus, our results suggest that many *in vivo *targets and much of the tissue-specific regulation by the Rb/E2F complex still remains to be discovered in mammalian systems.

### Tissue-specific relationships between LIN-35 and EFL-1/DPL-1

In many or most somatic tissues, LIN-35 and EFL-1/DPL-1 bind at many of the same targets. However, the tissue-specific binding profiles reveal that these factors do not always co-occupy the same binding sites, but exhibit uniquely bound sites distinct to particular cell types. The tissue-specific relationship between LIN-35 and EFL-1/DPL-1 binding correlates with effects on gene expression. In the germline, EFL-1 and DPL-1 frequently bind DNA in the absence of appreciable LIN-35 binding, and EFL-1/DPL-1 act independently of LIN-35 to promote expression. In the soma, EFL-1/DPL-1 targets exhibit extensive LIN-35 binding, and their expression is either inhibited or apparently unaffected by LIN-35 activity.

One possible mechanism for how LIN-35 might be specifically inhibited from binding in the germline comes from mammalian studies that have shown that Rb is largely refractory to ChIP analysis in transformed cells (reviewed in [34]). The phosphorylation status of Rb apparently alters its association with chromatin: phosphorylated Rb shows poor binding, while a phosphorylation-defective mutant has increased binding [35]. Therefore, one possibility is that post-translational regulation of LIN-35/Rb, perhaps by phosphorylation, limits its association with chromatin in a germline-specific manner. Because transformed cells and germline cells both represent undifferentiated cell types, the inability of LIN-35/Rb to effectively bind chromatin could be a general property of progenitor cells *in vivo*.

By contrast to the germline, many intestine-specific binding sites exhibited strong LIN-35 binding in the absence of substantial binding by EFL-1. Although two other E2F-like proteins are encoded in the *C. elegans *genome, neither appears functionally redundant with EFL-1. EFL-2 primarily has a role in regulating apoptosis [36], while F49E12.6 exhibits relatively poor binding by ChIP-seq and has little overlap with EFL-1 (data not shown). Moreover, very few genes in the intestine-specific set have an upstream consensus E2F sequence (unlike the other datasets), and the broad LIN-35 peaks are not restricted to promoter regions. All of these observations are consistent with the idea that LIN-35 can be recruited to multiple sites in the genome through a mechanism that does not depend on binding by an E2F-like protein.

The intestine-specific peaks are very broad and have a lower correlation with annotated genes compared to the narrow peaks typically produced by sequence-specific transcription factor binding. Moreover, the intestine-specific set is not enriched for germline-expressed genes, and even exhibits a preference for sites on the X chromosome, which is opposite to the trends for the other categories of binding sites. These intestine-specific sites occur primarily in the intestine, as they are much reduced when the entire soma is assayed. These specialized sites could be the means by which LIN-35 mediates certain intestine-specific functions, such as influencing endoreplication of intestinal nuclei [16] and guarding against a high temperature larval arrest [10]. Intriguingly, the heterochromatin-associated protein HPL-2 co-occupies these sites with LIN-35, and *hpl-2 *mutants exhibit similar endoreplication defects and a similar larval arrest as *lin-35 *mutants. These sites might mark some tissue-specific chromatin conformation that serves as a point of entry for the replication machinery and/or is permissive for germline gene expression.

### Key targets involved in the soma-to-germline transformation of *lin-35 *mutants

The identification of tissue-specific target genes sheds new light on the diverse mechanisms by which LIN-35, EFL-1 and DPL-1 influence the fate and function of different cell types. In the germline, a relatively straightforward relationship exists between target genes and the defects in oogenesis and early embryogenesis of *efl-1 *and *dpl-1 *mutants: EFL-1/DPL-1 directly bind at and promote the expression of many genes known to act in oogenesis and embryogenesis. The situation in the soma is more complex, at least in the L1 animals in which we analyzed binding profiles. Most somatic target genes directly bound by LIN-35, EFL-1, and DPL-1 have unaltered transcript levels in *lin-35 *mutants either by microarray or qRT-PCR analysis. The SynMuv A pathway is functionally redundant with the SynMuv B pathway, and its activity might compensate for certain phenotypes of *lin-35 *mutants, stabilizing expression of a subset of direct targets.

Strikingly, mutation of *lin-35 *results in substantial up-regulation of many genes that do not have LIN-35 binding nearby. How expression of these indirect targets is affected is unknown, but one direct target gene, *mes-4*, might link LIN-35 DNA binding with indirect effects on regulation of a subset of these genes. *mes-4 *encodes an H3K36 histone methyltransferase that preferentially acts on germline-expressed genes to promote their expression in the germline of the next generation [29], and its activity is essential for the soma-to-germline transformation of *lin-35 *mutants. MES-4 activity and LIN-35 activity could be in separate pathways that converge to oppositely regulate common gene targets, but our data suggest that their relationship is linear, at least in some tissues. LIN-35/EFL-1/DPL-1 binds to the promoter of *mes-4*, and limits its expression in at least a subset of somatic tissues. In *lin-35 *mutants, EFL-1 no longer binds the *mes-4 *locus, whereas most other EFL-1 binding sites persist, indicating that mutation of *lin-35 *disrupts the DRM complex more extensively at *mes-4 *than other direct target genes. Ectopically expressed MES-4 then inappropriately promotes somatic expression of germline-expressed genes. Ultimately, these upregulated genes in the soma are likely to mediate much of the conversion from somatic to germline characteristics.

Indeed, several indirect somatic targets were of particular interest in mediating this phenotype, such as those acting in germline-specific small RNA pathways. Strikingly, these genes are direct targets of EFL-1/DPL-1 in the germline but not in somatic tissues of wild-type animals. We wondered whether the EFL-1/DPL-1 binding sites utilized in the germline could become accessible to EFL-1/DPL-1 in the soma in the absence of LIN-35 activity, but found that EFL-1 is not recruited to these loci in *lin-35 *mutants (Figure S6C in Additional file 1), suggesting some other mechanism for their regulation. Potentially, MES-4 promotes their expression in *lin-35 *mutants instead.

Given the enhanced RNAi sensitivity of *lin-35 *mutants, we tested several of these small RNA pathway genes for a key role in the soma-to-germline transformation, and found that reduction of *wago-1 *or *csr-1 *activity suppressed the *lin-35 *larval arrest phenotype. WAGO-1 and CSR-1 bind to distinct pools of small 22G RNAs that target different classes of genes [32,33]. In particular, CSR-1-associated 22G RNAs match genes expressed in the germline. However, whether these 22G RNAs contribute to ectopic germline gene expression in the soma of *lin-35 *mutants still requires exploration. Intriguingly, reduction of both *csr-1 *and *mes-4 *activity simultaneously does not lead to greater suppression of the *lin-35 *larval arrest phenotype than either alone, and MES-4 and CSR-1-associated 22Gs appear to target largely overlapping sets of germline-expressed genes. Possibly, CSR-1 and MES-4 might cooperate to mark and promote the expression of germline genes in *lin-35 *mutants.

Thus, the tissue-specific binding profiles led to the implication of specific components of small RNA pathways as essential mediators of the soma-to-germline transformation of *lin-35 *mutants. Precedence exists for one or a few indirect target genes playing a key role in a prominent *lin-35 *mutant phenotype: de-regulation of a single target gene, *lin-3*, is sufficient to induce the multivulva phenotype of SynMuv mutants [37]. Strikingly, like *csr-1, lin-3 *also appears to be an indirect target of LIN-35 in the soma, at least in L1 animals (data not shown).

## Conclusions

We present the first in depth examination of tissue-specific binding by the Rb/E2F regulatory pathway *in vivo*. These data highlight unique and sometimes unexpected properties of this pathway in different tissues, and clearly demonstrate that Rb/E2F have specialized roles in both progenitor and differentiated cell types. Future studies should be directed toward investigating many of the individual gene regulatory events that could play key roles in mediating the tissue-specific phenotypes of this intriguing master regulator.

## Materials and methods

### Strain maintenance

Nematode strain maintenance was as described [38]. *C. elegans *strain N2 was used as the wild-type strain, in addition to the following variants: LGI, *lin-35(n745)*; LGII, *unc-4(e120), dpl-1(n3316) unc-4(e120)/*mnC1 *dpy-10(e128) unc-52(e444)) *(MT9940); LG III, *unc-119(ed3)*. All experiments were conducted at 20°C unless otherwise indicated.

### Transgene construction and analysis

Tissue-specific transgenes were constructed using the Multisite Gateway Cloning system (Invitrogen, Carlsbad, CA, USA). The upstream regulatory sequences from *lin-35, efl-1, dpl-1*, and *ges-1 *were cloned into pDONRP4P1R, and the *pie-1 *regulatory sequence in this vector (pCG142) was purchased (Addgene, Cambridge, MA, USA). The genomic sequences of *dpl-1, lin-35, efl-1 *and *hpl-2 *were amplified from N2 genomic DNA and cloned into pDONR201. The GFP:FLAG sequence was amplified from LIN-28::GFP:FLAG (a gift from Giovanni Stefani) and then PCR-stitched to the endogenous 3' UTR of each gene and cloned into pDONRP2RP3. All primer sequences are available upon request. Each entry clone was verified by sequencing before recombination into the destination vector, pCG150 (Addgene) using LR Clonase II Plus (Invitrogen). The resulting constructs, which contain an *unc-119 *rescue fragment, were then transformed into *unc-119(ed3) *worms using microparticle bombardment [39]. At least one independent, low copy number, integrated line was generated for each fusion construct. GFP expression of each construct was visualized using a Zeiss Axioplan with DIC and 488 wavelength for GFP. Images were collected using a Zeiss AxioCam MRm camera and processed using Axiovision software (Zeiss, Oberkochen, Germany). Supplemental Table 1 in Additional file 1 lists all strains used in this study.

### ChIP-seq

ChIP assays were conducted as previously described [12,14]. Worms were staged by bleaching and L1 starvation. The starved L1 larvae were placed on OP50 bacteria for 6 hours for L1 collection at 20°C, or for 4 hours for L1 collection at 26°C. Young adult collection was performed after 62 hours at 20°C. Samples were crosslinked with 2% formaldehyde for 30 minutes at room temperature and then quenched using 1 M Tris pH 7.5. The pelleted worms were then quick frozen in liquid nitrogen and stored at -80°C. Samples were sonicated to obtain 200 to 800 bp DNA fragments. For each sample, 2.2 mg of cell extract was immunoprecipitated using 7.5 μg of goat anti-GFP (gift from Tony Hyman), anti-IgG (R&D Systems, Minneapolis, MN, USA), or 5 μg anti-EFL-1 (Novus Biologicals, Littleton, CO, USA) antibodies. The enriched DNA fragments and input control (genomic DNA from same sample) were used for library preparation as previously described [12] in order to perform deep sequencing on the Illumina GA2 platform. A multiplex adaptor system was used to enable sequencing of four samples in each flow cell as previously described [40]. Table S2 in Additional file 1 contains the number of reads for each sample and replicate used in the analyses.

The raw data were processed as previously described [14]. Correlation analysis, peak calling and gene target assignment were also as previously described [14,19]. Briefly, for correlation analysis, we pooled raw signals from two biological replicates, normalized against input and used PeakSeq [19] to find peak regions of each factor from the pooled reads as well as for each replicate. Correlation between two biological replicates was determined by binning the binding peaks called by PeakSeq for each replicate from pooled reads (q-value cutoff of 0.001) into non-overlapped 100-nucleotide windows to avoid variation from peaks of different widths [19]. Raw reads at each window were counted from both replicates and used to calculate the Pearson correlation coefficient between replicates. Note that the number of binding sites ascribed to each tissue-specific dataset is not equivalent to the number of gene targets for that set. Some binding sites are not assigned to any target gene, and other binding sites are assigned to more than one candidate target. For instance, 415 germline-specific binding sites were assigned to 379 target genes. All ChIP-seq data have been deposited in Gene Expression Omnibus (GEO), under accession number GSE30246.

### Bioinformatic analysis of binding sites

To determine the functional categories of genes associated with each set of tissue-specific binding sites, we used DAVID [41] to assign GO terms to the genes. The 'molecular function' category from each tissue-specific dataset was sorted by significance and fold-enrichment as determined by DAVID, and redundant or overlapping categories were manually removed. The top ten categories with enrichment greater than two-fold and a *P *< 0.05 (modified Fisher exact test) were then graphed as in Figure 3e. The raw output for this analysis is provided in Additional file 5.

We compared genes known to be regulated in the germline based on [21] with each tissue-specific dataset. For this comparison, only the 'intrinsic' and 'oogenesis-enriched' genes, totaling 2,218 genes (approximately 10% of the total genes in the genome), were considered germline-expressed. Pearson's chi square analysis was performed to determine the significance of over-representation. To determine if bound genes were significantly over-represented among differentially regulated genes in previous microarray experiments, we collected lists of differentially regulated genes from [10,11,20] based on the criteria of each study. The tissue-specific gene targets were then compared with each list of differentially regulated genes for overlap. A hypergeometric probability test was utilized to determine the significance of the overlap [42].

### Gene expression analyses

qRT-PCR analysis was carried out as follows. Wild-type and *lin-35(n745) *L1-staged animals were grown at 20°C to the gravid adult stage and then bleached to isolate embryos. Embryos were cultured in S-basal at 26°C until the following day when they were placed on OP50 plates for 4 hours at 26°C until they were collected for RNA isolation. *unc-4 *control and *dpl-1 *mutant starved L1 animals were grown at 20°C for 72 hours on OP50 plates before dissected gonads were harvested. Adult worms were placed in dissection buffer (M9 with 0.1% levamisole and 0.001% Tween20) on a coverslip. We used 30 1/2 gauge needles to extrude approximately 112 gonad arms from each genotype, excising each just proximal to the spermatheca. Dissected gonads were carefully transferred to an eppendorf containing Trizol (Invitrogen). Total RNA from each sample (L1 animals and dissected gonads) was isolated using Trizol and then DNase treated with DNA-free (Ambion, Austin, TX, USA). Total RNA (250 ng) from each genotype was reverse transcribed using the Omniscript RT kit (Qiagen, Valencia, CA, USA). Gene-specific PCR was performed in duplicate for both RT and no RT conditions using the same protocol as ChIP-qPCR, or using Brilliant II SYBR Green QPCR Low Rox on the Stratagene Mx3000P system (Agilent Technologies, Santa Clara, CA, USA) with three-step cycling with an annealing temperature of 55°C followed by a dissociation program. Cycle threshold (Ct) values were normalized using primers specific to the housekeeping hexokinase gene, H25P06.1, or *act-3*.

*In situ *analysis was carried out according to [43] with modifications as described in [44]. Probes were prepared from partial cDNAs cloned in the pCR2.1 vector (Invitrogen). cDNA fragments used for *rme-2 *were nucleotides 1 to 1,029 and nucleotide 2,006 to 3,074 for *par-3 *where numbering begins at the ATG in the predicted spliced cDNA. Gonads were stained with BCIP/NBT tablets (Roche, Mannheim, Germany) for 3 hours for *rme-2 *and 5 hours for *par-3*, mounted, and viewed using a Zeiss Axioplan 2 imaging epifluorescence microscope.

The published microarray data used in this manuscript for comparison to the ChIP-seq data are in the GEO with accession numbers GSE26823-GSE26825 [10], GSE5071 [11], GSE6547 [20] and GSE715-GSE737 [21].

### RNAi and high-temperature arrest assay

RNAi by feeding was performed using clones from the Ahringer library [45]. Overnight cultures were grown at 37°C in Luria broth containing 50 μg/ml ampicillin and 12.5 μg/ml tetracycline and then a 5 hour culture was grown in Luria broth with 50 μg/ml ampicillin before plating on NGM plates containing 1 mM isopropyl β-D-1-thiogalactopyranoside (IPTG) and 50 μg/ml ampicillin. *lin-35(n745) *L4 animals were placed on feeding bacteria for at least 18 hours at 26°C and then transferred to fresh RNAi plates and allowed to lay embryos for 5 to 6 hours. Progeny were scored for larval arrest 3 to 4 days later. In order to bypass the embryonic lethality of *ego-1(RNAi)*, cultures were 1:1 diluted with L4440 empty vector.

## Abbreviations

bp: base pair; ChIP: chromatin immunoprecipitation; GEO: Gene Expression Omnibus; GFP: green fluorescent protein; GO: Gene Ontology; PCR: polymerase chain reaction; qPCR: quantitative PCR; RNAi: RNA interference; UTR: untranslated region.

## Competing interests

The authors declare that they have no competing interests.

## Authors' contributions

MK and VR conceived and designed the experiments. MK performed the experiments. MK and VR analyzed the data. VR and MK wrote the manuscript. WN helped with data analysis. ZL performed the target calling. GW helped in the generation of expression data. All authors read and approved the final manuscript.

## Supplementary Material

Additional file 1 **Supplementary figures and tables**. Figure S1: a diagram of each tissue-specific construct and expression of each transgenic strain as determined by GFP signal. Figure S2: demonstration that the GFP-tagged constructs rescue the mutant phenotypes of *dpl-1 *and *lin-35 *mutants. Figure S3: high correlation between replicates for each factor and between different experiments and transgenic EFL-1 binding mirrors endogenous binding in the L1 soma. Figure S4: Venn diagrams that show the comparison of called binding sites for each factor in each tissue between factors within a tissue and between tissues for a given factor. Figure S5: tissue-specific binding for a subset of germline-specific and soma-specific sites using ChIP-qPCR. Figure S6: an example of other direct target genes that still retain EFL-1 binding in *lin-35 *mutants. Additional binding profiles at the loci encoding various candidate small RNA pathway regulatory proteins not shown in Figure 5a. EFL-1 is not ectopically recruited to the promoters of germline-specific small RNA regulators. Figure S7: MEME analysis that shows that tissue-specific targets have distinct E2F binding motifs. Table S1: a list of all the strains used for ChIP-seq analyses. Table S2: number of reads for each sample and replicate used in the analyses. Also included is a section describing the materials and methods used for the additional data files. Click here for file

Additional file 2 **Supplementary file 1**. A list of binding sites for each factor in each tissue on a separate sheet. Click here for file

Additional file 3 **Supplementary file 2**. Target genes and intergenic binding sites for tissue-specific datasets. Click here for file

Additional file 4 **Supplementary file 3**. Targets overlapping with LIN-54 ChIP-chip study [15]. Click here for file

Additional file 5 **Supplementary file 4**. GO categories for each tissue-specific target gene set returned by DAVID, and summary page showing selected categories for Figure 3e. Click here for file

Additional file 6 **Supplementary file 5**. Genes regulated by microarray analyses with overlaps to tissue-specific binding sites, along with statistical analysis. Click here for file

Additional file 7 **Supplementary file 6**. Genes targeted by 22G RNAs [32] compared to MES-4 target genes [29]. Click here for file

Additional file 8 **Supplementary file 7**. Precise sequences associated with each motif identified by MEME for each set of tissue-specific binding sites. Click here for file

## References

[B1] Hanahan D Weinberg RA Hallmarks of cancer: the next generation. Cell 2011 14 646 674 10.1016/j.cell.2011.02.013 21376230

[B2] Chen HZ Tsai SY Leone G Emerging roles of E2Fs in cancer: an exit from cell cycle control. Nat Rev Cancer 2009 14 785 797 10.1038/nrc2696 19851314 PMC3616489

[B3] Xu X Bieda M Jin VX Rabinovich A Oberley MJ Green R Farnham PJ A comprehensive ChIP chip analysis of E2F1, E2F4, and E2F6 in normal and tumor cells reveals interchangeable roles of E2F family members. Genome Res 2007 14 1550 1561 10.1101/gr.6783507 17908821 PMC2045138

[B4] Lee BK Bhinge AA Iyer VR Wide-ranging functions of E2F4 in transcriptional activation and repression revealed by genome-wide analysis. Nucleic Acids Res 2011 14 3558 3573 10.1093/nar/gkq1313 21247883 PMC3089461

[B5] Ceol CJ Horvitz HR *dpl-1 *DP and *efl-1 *E2F act with *lin-35 *Rb to antagonize Ras signaling in *C. elegans *vulval development. Mol Cell 2001 14 461 473 10.1016/S1097-2765(01)00194-0 11463372

[B6] Page BD Guedes S Waring D Priess JR The *C. elegans *E2F-and DP-related proteins are required for embryonic asymmetry and negatively regulate Ras/MAPK signaling. Mol Cell 2001 14 451 460 11463371 10.1016/s1097-2765(01)00193-9

[B7] Fay DS Yochem J The SynMuv genes of *Caenorhabditis elegans *in vulval development and beyond. Dev Biol 2007 14 1 9 10.1016/j.ydbio.2007.03.016 17434473 PMC1955466

[B8] Wu X Shi Z Cui M Han M Ruvkun G Repression of germline RNAi pathways in somatic cells by retinoblastoma pathway chromatin complexes. PLoS Genet 2012 14 e1002542 10.1371/journal.pgen.1002542 22412383 PMC3297578

[B9] Wang D Kennedy S Conte D Kim JK Gabel HW Kamath RS Mello CC Ruvkun G Somatic misexpression of germline P granules and enhanced RNA interference in retinoblastoma pathway mutants. Nature 2005 14 593 597 10.1038/nature04010 16049496

[B10] Petrella LN Wang W Spike CA Rechtsteiner A Reinke V Strome S synMuv B proteins antagonize germline fate in the intestine and ensure *C. elegans *survival. Development 2011 14 1069 1079 10.1242/dev.059501 21343362 PMC3042865

[B11] Chi W Reinke V Promotion of oogenesis and embryogenesis in the *C. elegans *gonad by EFL-1/DPL-1 (E2F) does not require LIN-35 (pRB). Development 2006 14 3147 3157 10.1242/dev.02490 16854972

[B12] Zhong M Niu W Lu ZJ Sarov M Murray JI Janette J Raha D Sheaffer KL Lam HYK Preston E Slightham C Hillier LW Brock T Agarwal A Auerbach R Hyman AA Gerstein M Mango SE Kim SK Waterston RH Reinke V Snyder M Genome-wide identification of binding sites defines distinct functions for *Caenorhabditis elegans *PHA-4/FOXA in development and environmental response. PLoS Genet 2010 14 e1000848 10.1371/journal.pgen.1000848 20174564 PMC2824807

[B13] Gerstein MB Lu ZJ Van Nostrand EL Cheng C Arshinoff BI Liu T Yip KY Robilotto R Rechtsteiner A Ikegami K Alves P Chateigner A Perry M Morris M Auerbach RK Feng X Leng J Vielle A Niu W Rhrissorrakrai K Agarwal A Alexander RP Barber G Brdlik CM Brennan J Brouillet JJ Carr A Cheung M-S Clawson H Contrino S Integrative analysis of the *Caenorhabditis elegans *genome by the modENCODE Project. Science 2010 14 1775 1787 10.1126/science.1196914 21177976 PMC3142569

[B14] Niu W Lu ZJ Zhong M Sarov M Murray JI Brdlik CM Janette J Chen C Alves P Preston E Slightham C Jiang L Hyman AA Kim SK Waterston RH Gerstein M Snyder M Reinke V Diverse transcription factor binding features revealed by genome-wide ChIP-seq in *C. elegans*. Genome Res 2011 14 245 254 10.1101/gr.114587.110 21177963 PMC3032928

[B15] Tabuchi TM Deplancke B Osato N Zhu LJ Barrasa MI Harrison MM Horvitz HR Walhout AJM Hagstrom KA Chromosome-Biased Binding and Gene Regulation by the *Caenorhabditis elegans *DRM Complex. PLoS Genet 2011 14 e1002074 10.1371/journal.pgen.1002074 21589891 PMC3093354

[B16] Ouellet J Roy R The lin-35/Rb and RNAi pathways cooperate to regulate a key cell cycle transition in *C. elegans*. BMC Dev Biol 2007 14 38 10.1186/1471-213X-7-38 17466069 PMC1877806

[B17] Byerly L Cassada RC Russell RL The life cycle of the nematode *Caenorhabditis elegans** 1:: I. Wild-type growth and reproduction. Dev Biol 1976 14 23 33 10.1016/0012-1606(76)90119-6 988845

[B18] Sulston JE Schierenberg E White JG Thomson JN The embryonic cell lineage of the nematode *Caenorhabditis elegans*. Dev Biol 1983 14 64 119 10.1016/0012-1606(83)90201-4 6684600

[B19] Rozowsky J Euskirchen G Auerbach RK Zhang ZD Gibson T Bjornson R Carriero N Snyder M Gerstein MB PeakSeq enables systematic scoring of ChIP-seq experiments relative to controls. Nat Biotechnol 2009 14 66 75 10.1038/nbt.1518 19122651 PMC2924752

[B20] Kirienko NV Fay DS Transcriptome profiling of the *C. elegans *Rb ortholog reveals diverse developmental roles. Dev Biol 2007 14 674 684 10.1016/j.ydbio.2007.02.021 17368442 PMC2680605

[B21] Reinke V Gil IS Ward S Kazmer K Genome-wide germline-enriched and sex-biased expression profiles in *Caenorhabditis elegans*. Development 2004 14 311 14668411 10.1242/dev.00914

[B22] Reinke V Smith HE Nance J Wang J Van Doren C Begley R Jones SJ Davis EB Scherer S Ward S Kim SK A global profile of germline gene expression in *C. elegans*. Mol Cell 2000 14 605 616 10.1016/S1097-2765(00)00059-9 11030340

[B23] Kelly WG Schaner CE Dernburg AF Lee MH Kim SK Villeneuve AM Reinke V X-chromosome silencing in the germline of *C. elegans*. Development 2002 14 479 11807039 10.1242/dev.129.2.479 PMC4066729

[B24] Chi W Reinke V DPL-1 (DP) acts in the germ line to coordinate ovulation and fertilization in *C. elegans*. Mech Dev 2009 14 406 416 10.1016/j.mod.2009.01.008 19368797 PMC2680456

[B25] Dimova DK Dyson NJ The E2F transcriptional network: old acquaintances with new faces. Oncogene 2005 14 2810 2826 10.1038/sj.onc.1208612 15838517

[B26] Lin B Reinke V The candidate MAP kinase phosphorylation substrate DPL-1 (DP) promotes expression of the MAP kinase phosphatase LIP-1 in *C. elegans *germ cells. Dev Biol 2008 14 50 61 10.1016/j.ydbio.2007.12.042 18304523 PMC2359152

[B27] Farnham PJ Insights from genomic profiling of transcription factors. Nat Rev Genet 2009 14 605 616 10.1038/nrg2636 19668247 PMC2846386

[B28] Polley SRG Fay DS A network of genes antagonistic to the LIN-35 retinoblastoma protein of *Caenorhabditis elegans*. Genetics 2012 14 1367 1380 10.1534/genetics.112.140152 22542970 PMC3416014

[B29] Rechtsteiner A Ercan S Takasaki T Phippen TM Egelhofer TA Wang W Kimura H Lieb JD Strome S The histone H3K36 methyltransferase MES-4 acts epigenetically to transmit the memory of germline gene expression to progeny. PLoS Genet 2010 14 ii e1001091 10.1371/journal.pgen.1001091 PMC2932692 20824077

[B30] Grishok A Hoersch S Sharp PA RNA interference and retinoblastoma-related genes are required for repression of endogenous siRNA targets in *Caenorhabditis elegans*. Proc Natl Acad Sci USA 2008 14 20386 20391 10.1073/pnas.0810589105 19073934 PMC2629315

[B31] She X Xu X Fedotov A Kelly WG Maine EM Regulation of heterochromatin assembly on unpaired chromosomes during *Caenorhabditis elegans *meiosis by components of a small RNA-mediated pathway. PLoS Genet 2009 14 e1000624 10.1371/journal.pgen.1000624 19714217 PMC2726613

[B32] Claycomb JM Batista PJ Pang KM Gu W Vasale JJ Van Wolfswinkel JC Chaves DA Shirayama M Mitani S Ketting RF Conte D Jr Mello CC The Argonaute CSR-1 and its 22G-RNA cofactors are required for holocentric chromosome segregation. Cell 2009 14 123 134 10.1016/j.cell.2009.09.014 19804758 PMC2766185

[B33] Gu W Shirayama M Conte D Jr Vasale J Batista PJ Claycomb JM Moresco JJ Youngman EM Keys J Stoltz MJ Chen C-CG Chaves DA Duan S Kasschau KD Fahlgren N Yates JR Mitani S Carrington JC Mello CC Distinct Argonaute-mediated 22G-RNA pathways direct genome surveillance in the *C. elegans *germline. Mol Cell 2009 14 231 244 10.1016/j.molcel.2009.09.020 19800275 PMC2776052

[B34] Rizzolio F Esposito L Muresu D Fratamico R Jaraha R Caprioli GV Giordano A RB gene family: Genome-wide ChIP approaches could open undiscovered roads. J Cell Biochem 2009 14 839 843 10.1002/jcb.22448 20052675

[B35] Stengel KR Thangavel C Solomon DA Angus SP Zheng Y Knudsen ES Retinoblastoma/p107/p130 pocket proteins: protein dynamics and interactions with target gene promoters. J Biol Chem 2009 14 19265 19271 10.1074/jbc.M808740200 19279001 PMC2740551

[B36] Schertel C Conradt B *C. elegans *orthologs of components of the RB tumor suppressor complex have distinct pro-apoptotic functions. Development 2007 14 3691 3701 10.1242/dev.004606 17881492

[B37] Cui M Chen J Myers TR Hwang BJ Sternberg PW Greenwald I Han M SynMuv genes redundantly inhibit lin-3/EGF expression to prevent inappropriate vulval induction in *C. elegans*. Dev Cell 2006 14 667 672 10.1016/j.devcel.2006.04.001 16678779

[B38] Brenner S The Genetics of *Caenorhabditis elegans*. Genetics 1974 14 71 94 4366476 10.1093/genetics/77.1.71 PMC1213120

[B39] Praitis V Casey E Collar D Austin J Creation of low-copy integrated transgenic lines in *Caenorhabditis elegans*. Genetics 2001 14 1217 1226 11238406 10.1093/genetics/157.3.1217 PMC1461581

[B40] Lefrançois P Euskirchen GM Auerbach RK Rozowsky J Gibson T Yellman CM Gerstein M Snyder M Efficient yeast ChIP-Seq using multiplex short-read DNA sequencing. BMC genomics 2009 14 37 10.1186/1471-2164-10-37 19159457 PMC2656530

[B41] DAVID. http://david.abcc.ncifcrf.gov/

[B42] Statistical significance of the overlap between two groups of genes. http://nemates.org/MA/progs/overlap_stats.html

[B43] Jones AR Francis R Schedl T GLD-1, a cytoplasmic protein essential for oocyte differentiation, shows stage-and sex-specific expression during *Caenorhabditis elegans *germline development. Dev Biol 1996 14 165 183 10.1006/dbio.1996.0293 8948583

[B44] Leacock SW Reinke V Expression profiling of MAP kinase-mediated meiotic progression in *Caenorhabditis elegans*. PLoS Genet 2006 14 e174 10.1371/journal.pgen.0020174 17096596 PMC1635537

[B45] Fraser AG Kamath RS Zipperlen P Martinez-Campos M Sohrmann M Ahringer J Functional genomic analysis of *C. elegans *chromosome I by systematic RNA interference. Nature 2000 14 325 330 10.1038/35042517 11099033

