# Supplementary material for: Tissue-specific direct targets of Caenorhabditis elegans Rb/E2F dictate distinct somatic and germline programs
Source: Genome Biol. 2013 Jan 23;14(1):R5. doi: 10.1186/gb-2013-14-1-r5 (PMC4053757; doi:10.1186/gb-2013-14-1-r5)
Supplement: Additional file 1 — Supplementary figures and tables. Figure S1: a diagram of each tissue-specific construct and expression of each transgenic strain as determined by GFP signal. Figure S2: demonstration that the GFP-tagged constructs rescue the mutant phenotypes of dpl-1 and lin-35 mutants. Figure S3: high correlation between replicates for each factor and between different experiments and transgenic EFL-1 binding mirrors endogenous binding in the L1 soma. Figure S4: Venn diagrams that show the comparison of called binding sites for each factor in each tissue between factors within a tissue and between tissues for a given factor. Figure S5: tissue-specific binding for a subset of germline-specific and soma-specific sites using ChIP-qPCR. Figure S6: an example of other direct target genes that still retain EFL-1 binding in lin-35 mutants. Additional binding profiles at the loci encoding various candidate small RNA pathway regulatory proteins not shown in Figure 5a. EFL-1 is not ectopically recruited to the promoters of germline-specific small RNA regulators. Figure S7: MEME analysis that shows that tissue-specific targets have distinct E2F binding motifs. Table S1: a list of all the strains used for ChIP-seq analyses. Table S2: number of reads for each sample and replicate used in the analyses. Also included is a section describing the materials and methods used for the additional data files. [file gb-2013-14-1-r5-S1.PDF]

Additional data files

Kudron et al., Tissue-specific direct targets of *Caenorhabditis elegans* Rb/E2F dictate distinct somatic and germline programs

Additional file 1:

Supplemental figure legends

Supplemental Figure 1. Tissue-specific promoters drive expression in the germ line, ubiquitously in the soma, and in the intestine. A. Diagram of each tissue-specific construct. Different promoters were used to drive expression of genomic fragments containing *efl-1, dpl-1, lin-35* and *hpl-2* (black) in specific tissues (*pie-1* - germ line, endogenous – multiple tissues, and *ges-1* – intestine (grey)). Each coding sequence had a GFP:FLAG epitope fused in frame at the C-terminus (green), upstream of the native 3' UTR of each gene. **B.** Tissue-specific expression of transgenes as determined by GFP signal. Germline-specific expression of EFL-1, DPL-1 and LIN-35 using the PIE-1 promoter, and LIN-35 with the MEX-5 promoter is indicated in adult. Endogenous expression of EFL-1, DPL-1 and LIN-35 is shown in L1 animals. Intestine-specific expression of EFL-1, DPL-1, LIN-35 and HPL-2 is indicated in L1 animals. Note nuclear localization. Scale bar = 20 μm.

Supplemental Figure 2. GFP-tagged constructs rescue the mutant phenotypes of *dpl-1* and *lin-35* mutants. A. DIC and GFP images of *dpl-1(n3316)/*mnC1 heterozygotes (top), *dpl-1(n3316)* homozygotes (middle), and *dpl-1(n3316)* homozygotes carrying the germline DPL-1:GFP transgene (bottom) are shown. The distal tip is marked by an asterisk; o = immature oocytes, emo = endomitotic oocytes, e = embryos, s = spermatheca, v = vulva. Note the presence of embryos in *dpl-1(n3316)* mutants carrying the germline DPL-1:GFP transgenic

strain and the lack of endomitotic ooctyes (n≥17). **B-D.** Brood size assays were conducted for *lin-35* mutants carrying the endogenous LIN-35:GFP transgene (**B**), the germline LIN-35:GFP transgene (**C**), and the pMEX-5:LIN-35:GFP transgene (**D**). The number of progeny in *unc-119* mutants and *lin-35; unc-119* mutants are shown as controls. The brood size of *lin-35; unc-119* mutants carrying each transgene was significantly higher than *lin-35; unc-119* mutants alone, * = $p<0.001$. The *unc-119*-associated brood size defects are rescued because the transgene contains a rescuing copy of *unc-119*. Error bars indicate standard deviation (n ≥ 7). **E.** The percentage of animals arrested as larvae at 26°C in *unc-119* mutants, *lin-35; unc-119* mutants, *lin-35; unc-119* mutants carrying the endogenous LIN-35:GFP transgene and *lin-35; unc-119* mutants carrying the germline LIN-35:GFP transgene are shown.

Supplemental Figure 3. Replicates for each factor and between different experiments exhibit high overlap. A. Only peaks with a q value < 0.001 were included in the analysis. For each factor and between different experiments, the overlap (<1 nt) of called binding sites between the top 40% of each replicate/experiment was determined and graphed. **B.** Example of an ~1.2 Mb genomic region on Chr IV showing the similarity of binding between transgenic EFL-1 (Pefl-1:EFL-1 (anti-GFP)) in blue and endogenous EFL-1 (wild-type (anti-EFL-1)) in magenta. An input control is shown for each in black. An individual gene (*fem-3*) is shown below.

Supplemental Figure 4. Overlap of binding profiles between factors and across tissues. Called binding sites for each factor in each tissue were compared for overlap, between factors within a tissue (A) and between tissues for a given factor (B), and displayed as Venn diagrams. Venn diagrams are hand-drawn to approximate the relative levels of overlap and are not meant to be precise.

Supplemental Figure 5. Confirmation of tissue-specific binding for a subset of germline-specific and soma-specific sites by ChIP-qPCR. Average fold enrichment of binding from two technical replicates was determined for each gene using ChIP DNA from germline DPL-1 (red bars) or somatic DPL-1 (blue bars) compared to IgG control. Germline-specific targets and soma-specific targets are indicated with the black line. Error bars indicate standard error.

Supplemental Figure 6. EFL-1 is not ectopically recruited to the promoters of germline-specific small RNA regulators. **A.** Binding profiles of EFL-1 in the germ line (red) and soma (blue) using the GFP antibody and wild type (magenta) and *lin-35* (cyan) using the EFL-1 antibody at the *daf-14* locus as an example of most other direct target genes that still retain EFL-1 binding in *lin-35* mutants. An input control for each is shown below in black. **B.** Binding profiles at the *ego-1* and *wago-2* loci. The key for each factor and tissue is to the left of the tracks. One track is shown for each factor in sets corresponding to each tissue, with a control (input) sample for each tissue below (black). Red = germline-specific promoter, Blue = soma-specific promoter, Orange = intestine-specific promoter. Note the germline-specific binding at these loci. **C.** Binding profile of EFL-1 as described in **A** at the *wago-1* and *csr-1* loci. An input control for each is shown below in black. Note that there is only binding in the germ line and that these promoters are not occupied by EFL-1 in the soma of *lin-35* mutants.

Supplemental Figure 7. Tissue-specific targets have distinct E2F binding motifs. Consensus motifs derived from MEME analysis, including number of binding sites containing each motif, and the assigned E value, an estimate of how likely a similar motif would be found in random sequence. The motif labeled "germline E2F" most closely matches the motif found in Chi and Reinke (2006), while the motif labeled "somatic E2F" matches the motif in Kirienko and Fay (2007). Note that the germline E2F motif has a much stronger CCTT motif than the somatic E2F motif.

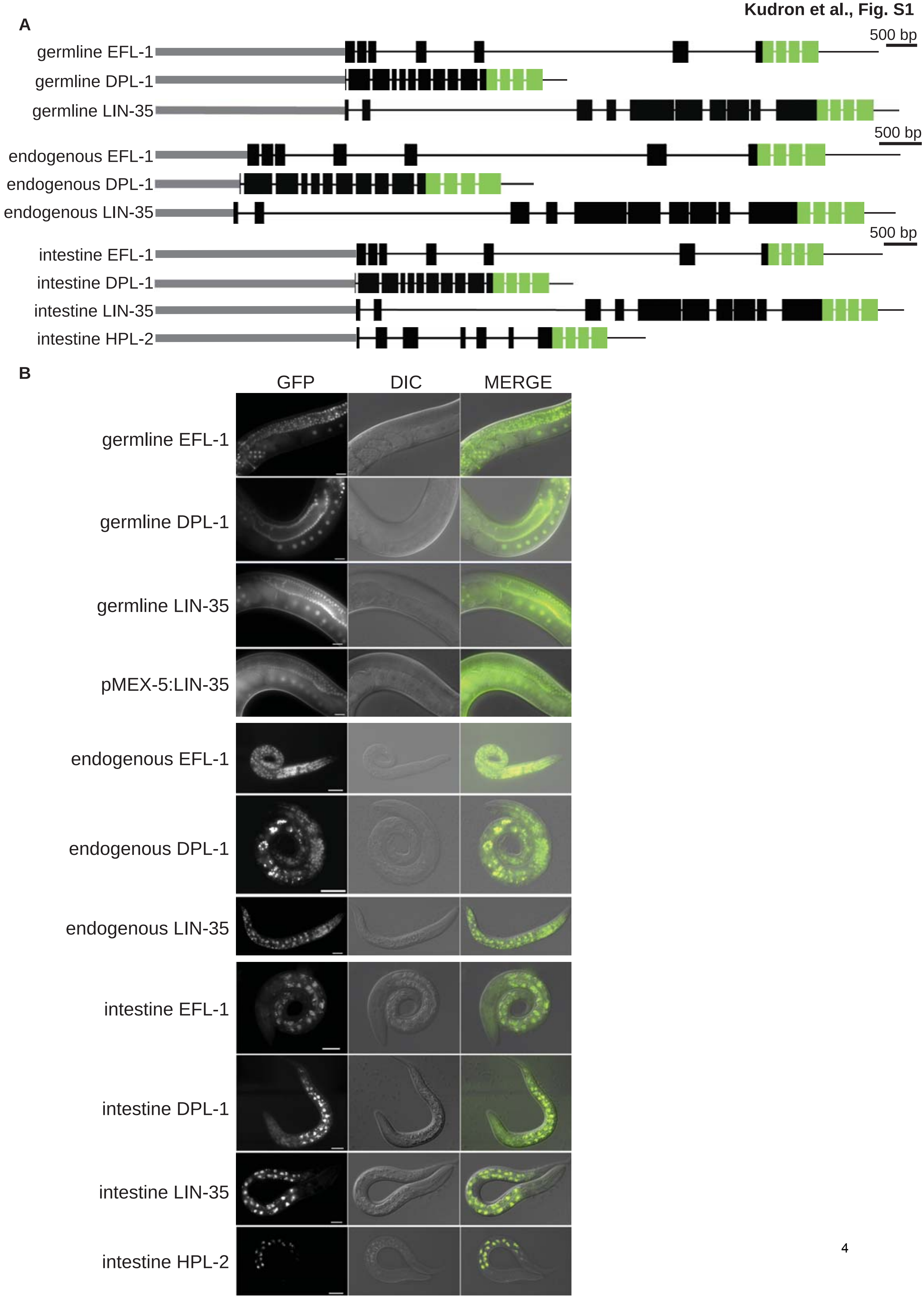
A
500 bp
germline EFL-1
germline DPL-1
germline LIN-35
500 bp
endogenous EFL-1
endogenous DPL-1
endogenous LIN-35
500 bp
intestine EFL-1
intestine DPL-1
intestine LIN-35
intestine HPL-2
B
GFP
DIC
MERGE
germline EFL-1
germline DPL-1
germline LIN-35
pMEX-5:LIN-35
endogenous EFL-1
endogenous DPL-1
endogenous LIN-35
intestine EFL-1
intestine DPL-1
intestine LIN-35
intestine HPL-2

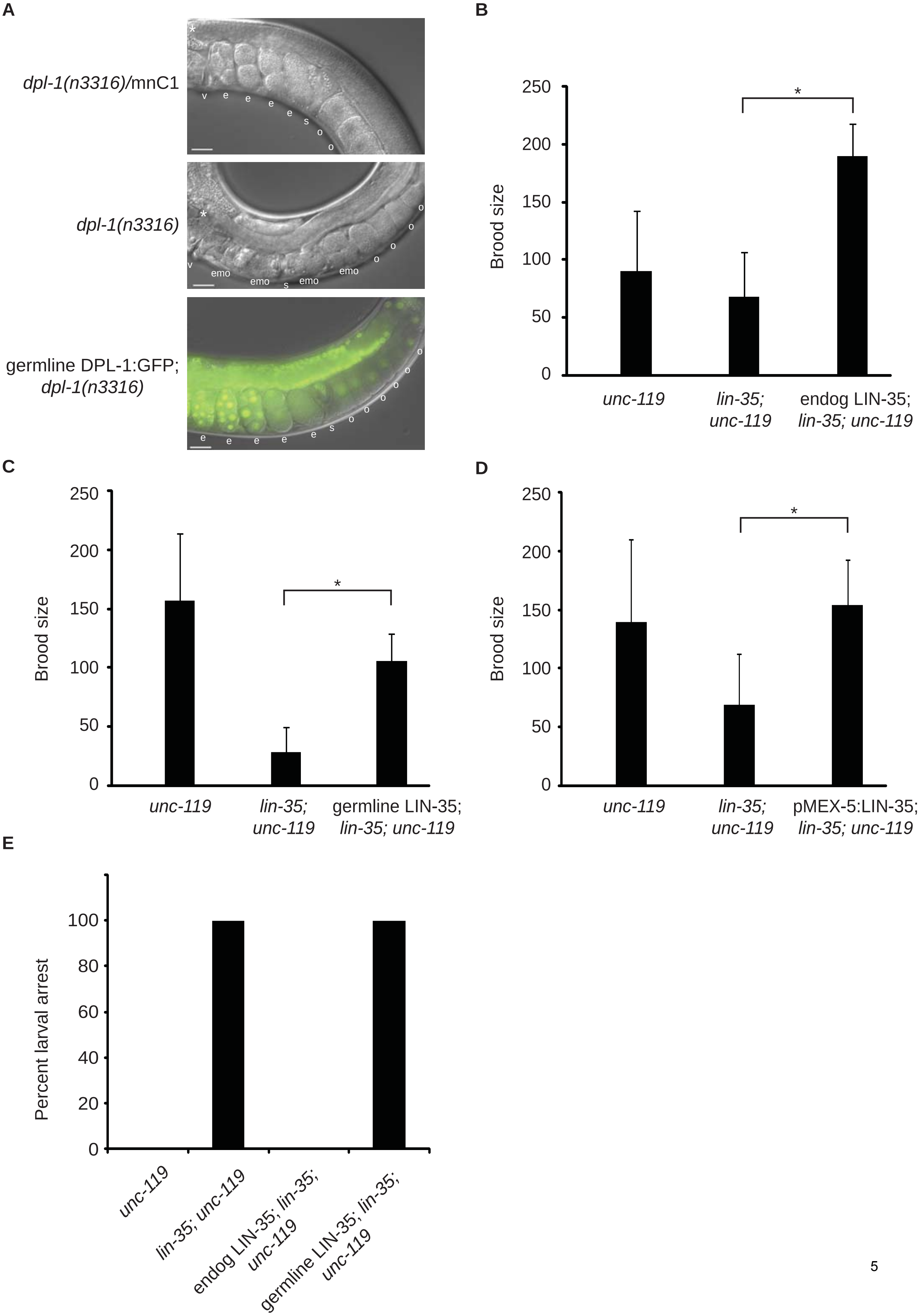

A
dpl-1(n3316)/mnC1
v e e e e s o o
dpl-1(n3316)
v emo emo s emo emo o o o o
germline DPL-1:GFP;
dpl-1(n3316)
e e e e e s o o o o o o o
B
Brood size
250
200
150
100
50
0
*
unc-119
lin-35; unc-119
endog LIN-35; lin-35; unc-119
C
Brood size
250
200
150
100
50
0
*
unc-119
lin-35; unc-119
germline LIN-35; lin-35; unc-119
D
Brood size
250
200
150
100
50
0
*
unc-119
lin-35; unc-119
pMEX-5:LIN-35; lin-35; unc-119
E
Percent larval arrest
100
80
60
40
20
0
unc-119
lin-35; unc-119
endog LIN-35; lin-35; unc-119
germline LIN-35; lin-35; unc-119

A

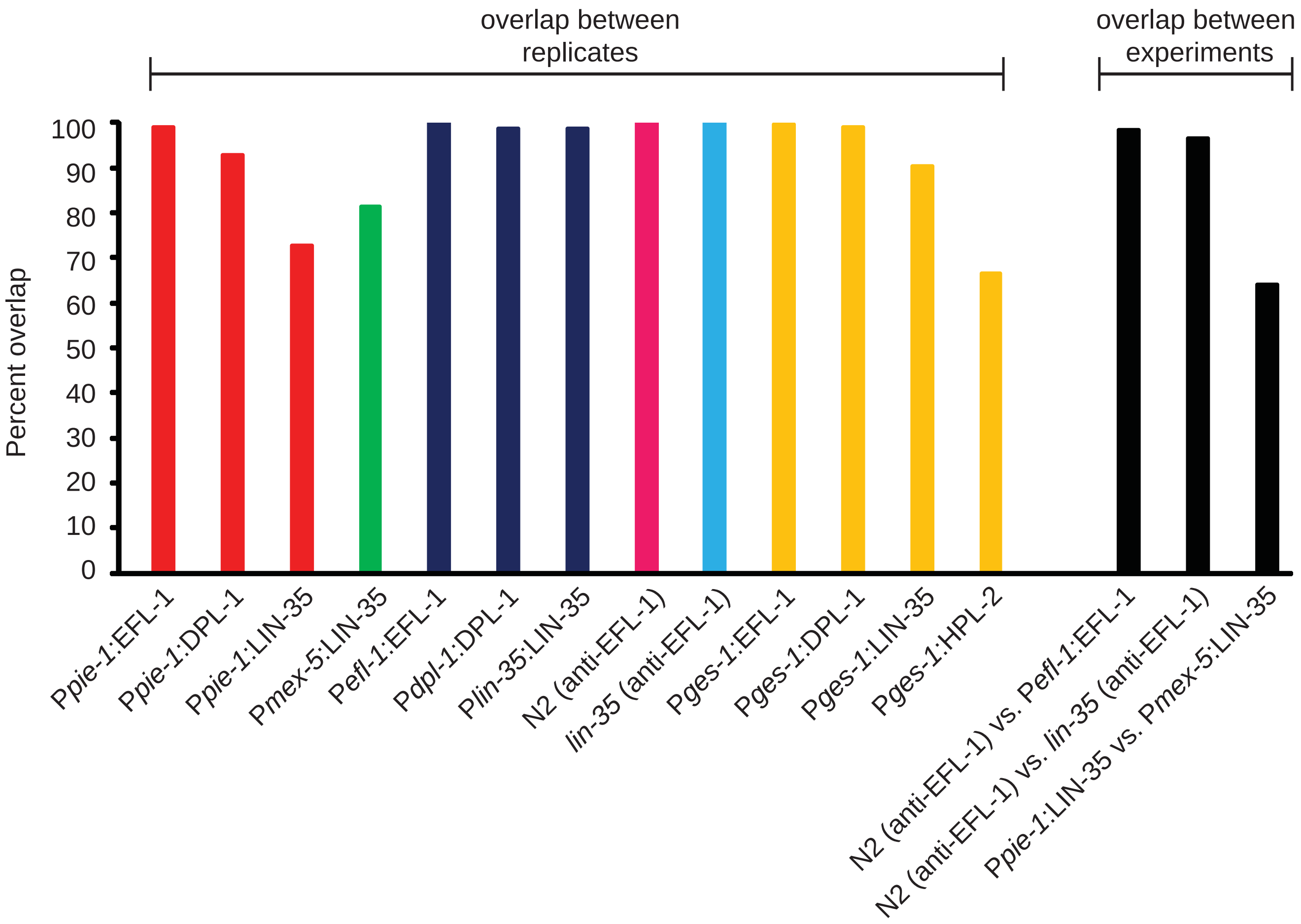
overlap between replicates
overlap between experiments
Percent overlap
100
90
80
70
60
50
40
30
20
10
0
Ppie-1:EFL-1
Ppie-1:DPL-1
Ppie-1:LIN-35
Pmex-5:LIN-35
Pefl-1:EFL-1
Pdpl-1:DPL-1
Plin-35:LIN-35
N2 (anti-EFL-1)
lin-35 (anti-EFL-1)
Pges-1:EFL-1
Pges-1:DPL-1
Pges-1:LIN-35
Pges-1:HPL-2
N2 (anti-EFL-1) vs. Pefl-1:EFL-1
N2 (anti-EFL-1) vs. lin-35 (anti-EFL-1)
Ppie-1:LIN-35 vs. Pmex-5:LIN-35

B

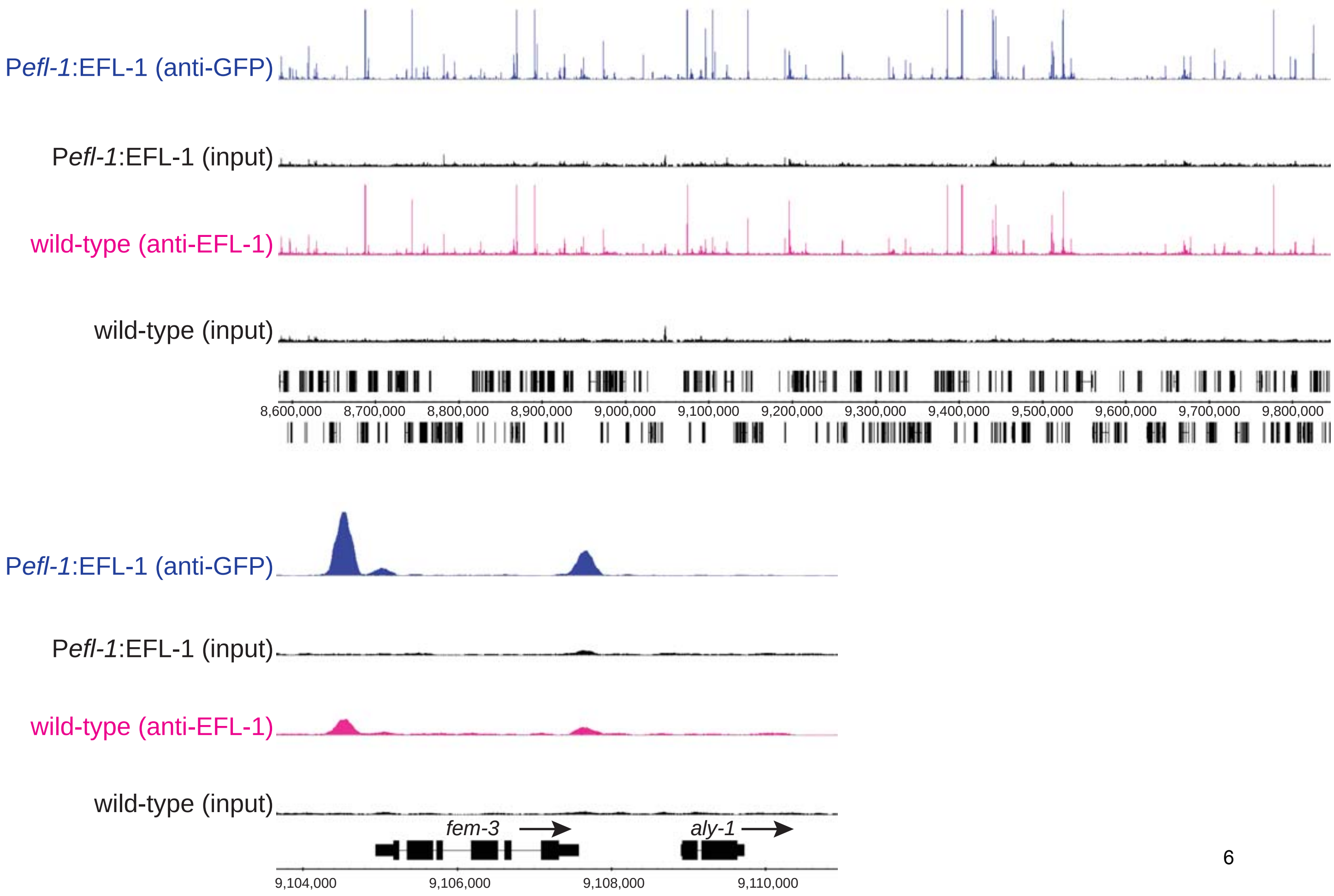
Pefl-1:EFL-1 (anti-GFP)
Pefl-1:EFL-1 (input)
wild-type (anti-EFL-1)
wild-type (input)
8,600,000
8,700,000
8,800,000
8,900,000
9,000,000
9,100,000
9,200,000
9,300,000
9,400,000
9,500,000
9,600,000
9,700,000
9,800,000
Pefl-1:EFL-1 (anti-GFP)
Pefl-1:EFL-1 (input)
wild-type (anti-EFL-1)
wild-type (input)
fem-3
aly-1
9,104,000
9,106,000
9,108,000
9,110,000

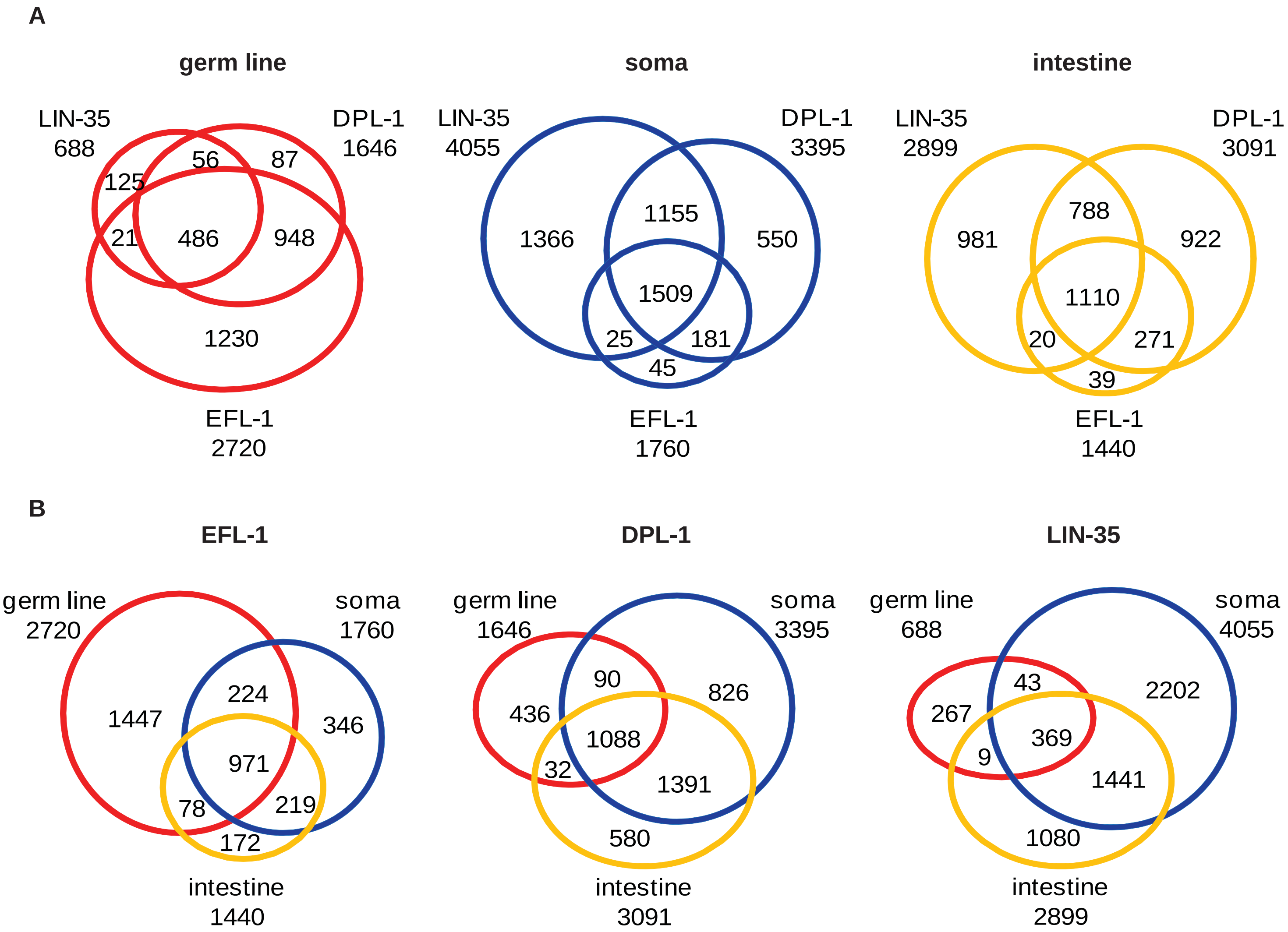
A
germ line
LIN-35
688
DPL-1
1646
125
56
87
21
486
948
1230
EFL-1
2720
soma
LIN-35
4055
DPL-1
3395
1366
1155
550
1509
25
181
45
EFL-1
1760
intestine
LIN-35
2899
DPL-1
3091
981
788
922
1110
20
271
39
EFL-1
1440
B
EFL-1
germ line
2720
soma
1760
1447
224
346
971
78
219
172
intestine
1440
DPL-1
germ line
1646
soma
3395
436
90
826
1088
32
1391
580
intestine
3091
LIN-35
germ line
688
soma
4055
267
43
2202
369
9
1441
1080
intestine
2899

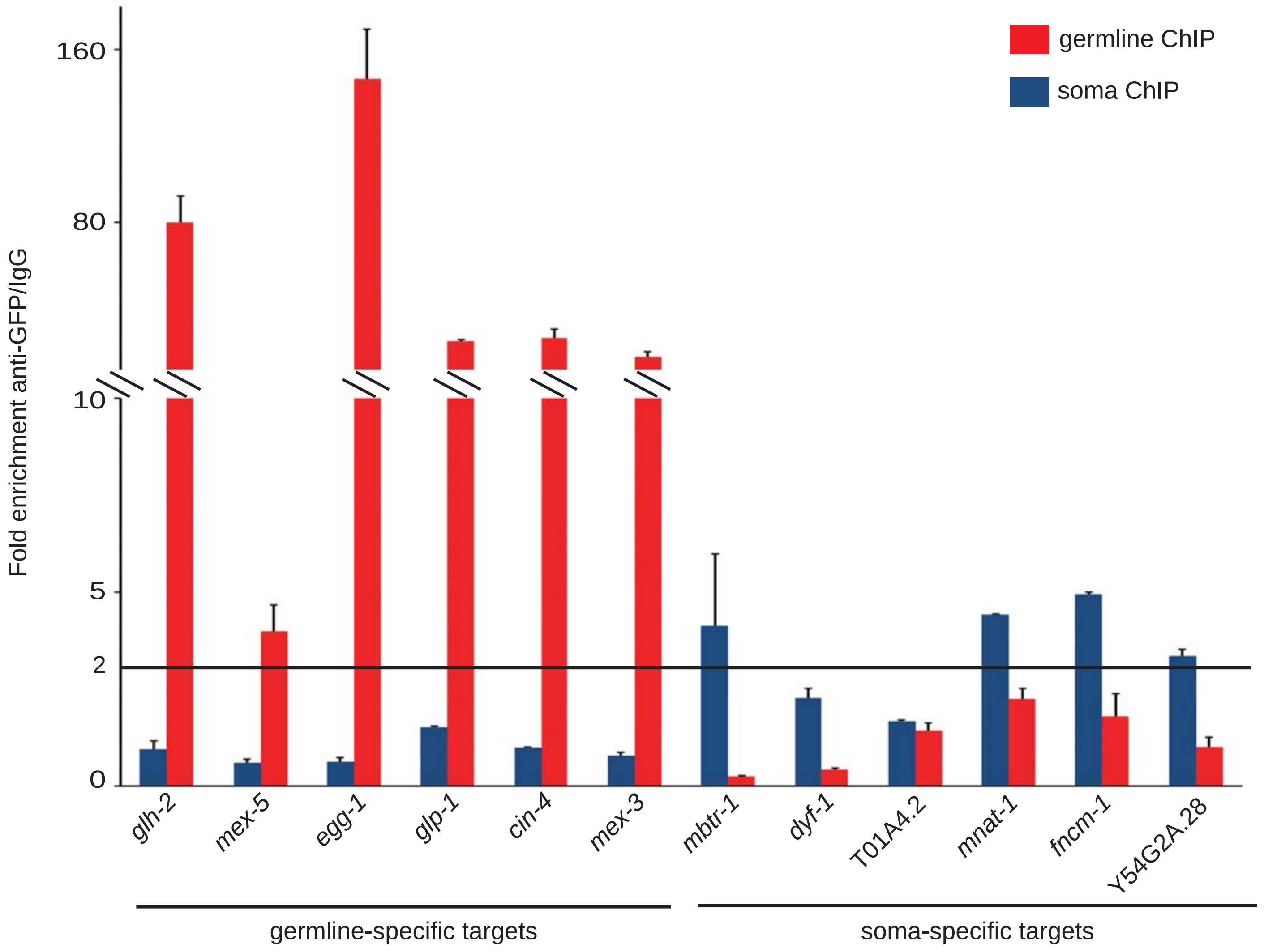
germline ChIP
soma ChIP
Fold enrichment anti-GFP/IgG
160
80
10
5
2
0
glh-2
mex-5
egg-1
glp-1
cin-4
mex-3
mbtr-1
dyf-1
T01A4.2
mnat-1
fncm-1
Y54G2A.28
germline-specific targets
soma-specific targets

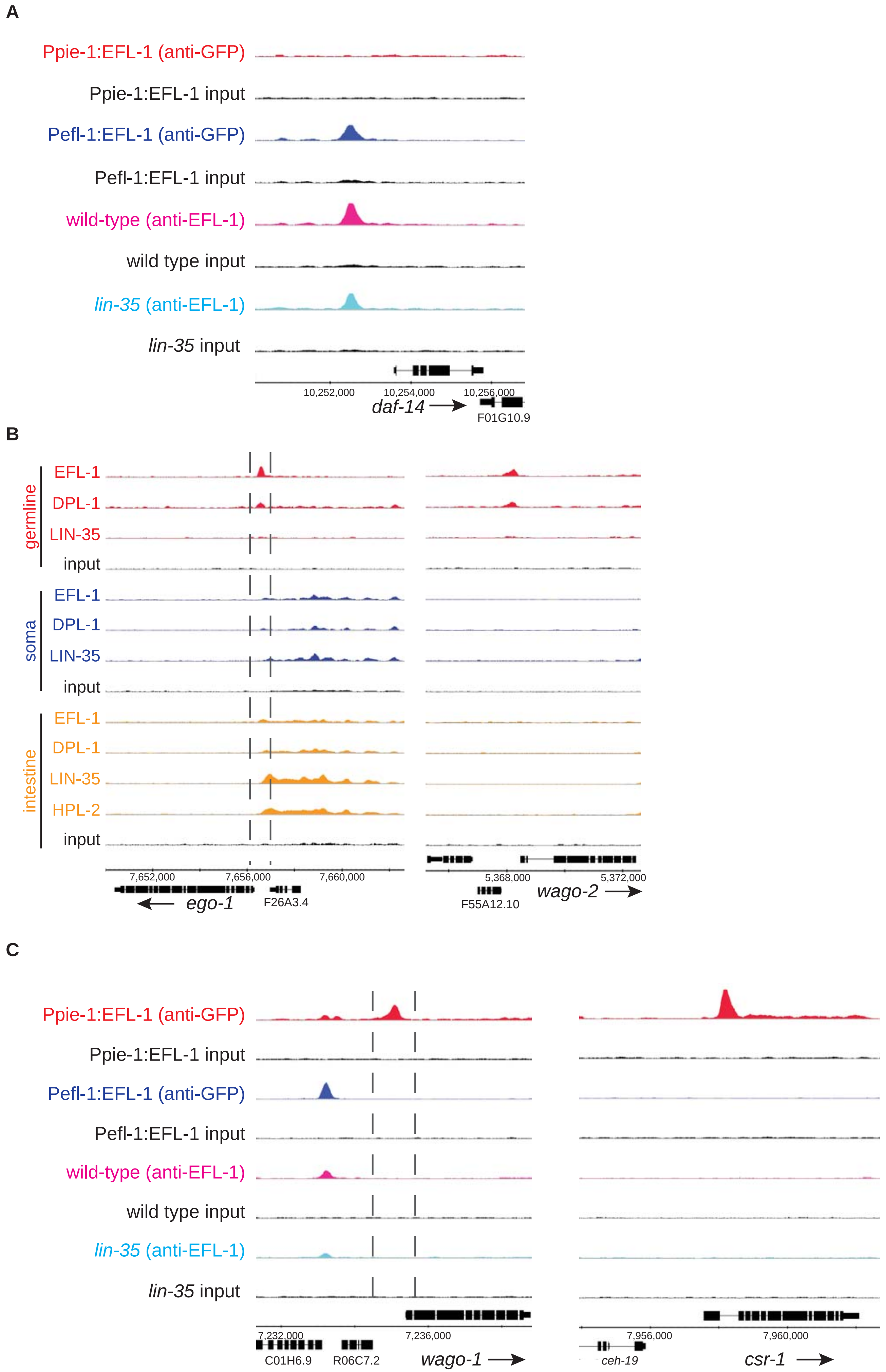
A
Ppie-1:EFL-1 (anti-GFP)
Ppie-1:EFL-1 input
Pefl-1:EFL-1 (anti-GFP)
Pefl-1:EFL-1 input
wild-type (anti-EFL-1)
wild type input
lin-35 (anti-EFL-1)
lin-35 input
10,252,000
10,254,000
10,256,000
daf-14
F01G10.9
B
germline
soma
intestine
EFL-1
DPL-1
LIN-35
HPL-2
input
7,652,000
7,656,000
7,660,000
ego-1
F26A3.4
5,368,000
5,372,000
F55A12.10
wago-2
C
Ppie-1:EFL-1 (anti-GFP)
Ppie-1:EFL-1 input
Pefl-1:EFL-1 (anti-GFP)
Pefl-1:EFL-1 input
wild-type (anti-EFL-1)
wild type input
lin-35 (anti-EFL-1)
lin-35 input
7,232,000
7,236,000
C01H6.9
R06C7.2
wago-1
7,956,000
7,960,000
ceh-19
csr-1

germline-specific

novel A
53 genes
E=1.0e^{-109}

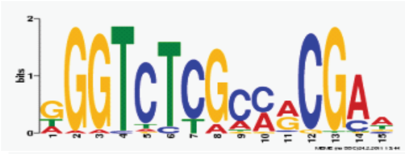

T-blocks
103 genes
E=7.3e^{-66}

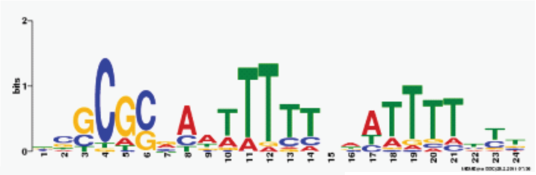

germline E2F
40 genes
E=2.3e^{-53}

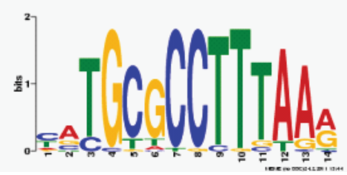

soma-specific

somatic E2F
53 genes
E=2.6e^{-167}

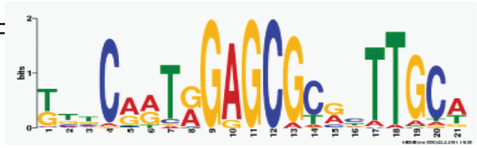

FOXO
34 genes
E=5.9e^{-36}

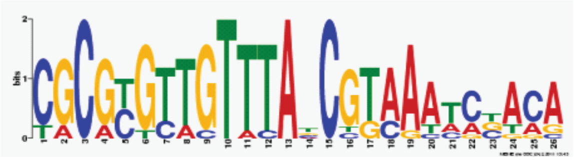

novel B
12 genes
E=2.2e^{-27}

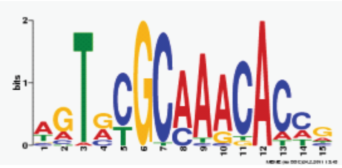

intestine-specific

novel C
83 genes
E=4.1e^{-70}

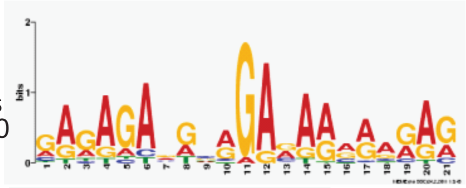

GATA
31 genes
E=1.9e^{-15}

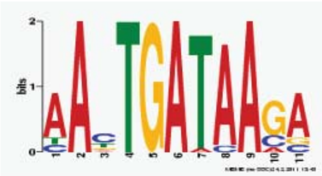

somatic E2F
13 genes
E=3.4e^{-07}

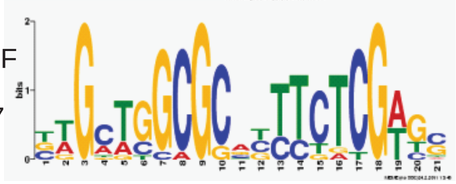

broadly-bound

somatic E2F
153 genes
E=1.6e^{-405}

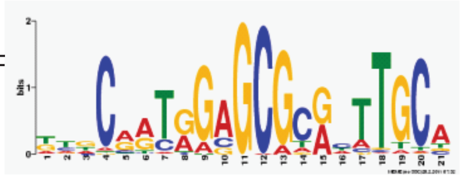

novel A
60 genes
E=1.8e^{-147}

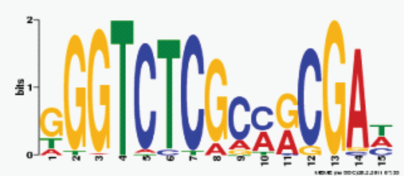

T-blocks
293 genes
E=2.2e^{-142}

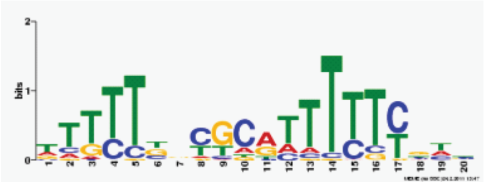

Supplemental Tables

Supplemental Table 1: Strain names and genotypes of tissue-specific strains used for ChIP-seq.

Tissue-specific construct	Strain name	Genotype
germline EFL-1	YL445	*unc119(ed3)* III; vrIs81 [pPIE-1::EFL-1::GFP FLAG: EFL-1 3'UTR, unc-119 (+)]
germline DPL-1	YL390	*unc119(ed3)* III; vrIs48 [pPIE-1::DPL-1::GFP FLAG: DPL-1 3'UTR, unc-119 (+)]
germline LIN-35	YL402	*unc119(ed3)* III; vrIs56[pPIE-1::LIN-35::GFP FLAG: LIN-35 3'UTR, unc-119 (+)]
germline (pMEX-5) LIN-35	YL468	*unc-119(ed3)* III; vrIs93 [pMEX-5::LIN-35:GFP:FLAG::LIN-35 3'UTR, unc-119 (+)])
endogenous EFL-1	YL424	*unc-119(ed3)* III; vrIs68[pEFL-1::EFL-1::GFP FLAG: EFL-1 3'UTR, unc-119 (+)]
endogenous DPL-1	YL425	*unc-119(ed3)* III; vrIs69[pDPL-1::DPL-1::GFP FLAG: DPL-1 3'UTR, unc-119 (+)]
endogenous LIN-35	YL409	*unc-119(ed3)* III; vrIs60 [pLIN-35::LIN-35:GFP:FLAG::LIN-35 3'UTR, unc-119 (+)])
intestine EFL-1	YL418	*unc-119(ed3)* III; vrIs65[pGES-1::EFL-1::GFP FLAG:EFL-1 3'UTR, unc-119 (+)]
intestine DPL-1	YL448	*unc-119(ed3)* III; vrIs83 [pGES-1::DPL-1::GFP FLAG: DPL-1 3'UTR, unc-119 (+)]
intestine LIN-35	YL398	*unc119(ed3)* III; vrIs55[pGES-1::LIN-35::GFP FLAG: LIN-35 3'UTR, unc-119 (+)]
intestine HPL-2	YL416	*unc119(ed3)* III; vrIs64[pGES-1::HPL-2::GFP FLAG: HPL-2 3'UTR, unc-119 (+)]

Supplemental Table 2. Number of reads for each sample and replicate used in the analyses.

Dataset name	Replicate number	Antibody/Input	Reads (x 10,000)
germline EFL-1	2	GFP	191
	3*	GFP	267
	2	Input	264
	3*	Input	393
germline DPL-1	1	GFP	113
	4*	GFP	125
	1	Input	211
	4	Input	261
germline LIN-35	2	GFP	139
	3*	GFP	232
	2	Input	346
	3	Input	380
germline (pMEX-5) LIN-35	1*	GFP	160
	2	GFP	65
	1	Input	698
	2	Input	386
endogenous EFL-1	1	GFP	360
	2*	GFP	276
	1	Input	232
	2*	Input	435
wild-type (anti-EFL-1)	1	EFL-1	461
	3*	EFL-1	576
	1	Input	682
	3*	Input	606
lin-35 (anti-EFL-1)	2*	EFL-1	528
	3	EFL-1	422
	2*	Input	495
	3	Input	565
endogenous DPL-1	1	GFP	192
	2*	GFP	227
	1	Input	364
	2	Input	170
endogenous LIN-35	1	GFP	218
	2*	GFP	171
	1	Input	307
	2	Input	182
intestine EFL-1	2*	GFP	161
	3	GFP	116
	2*	Input	106
	3	Input	112
intestine DPL-1	1*	GFP	440
	2	GFP	119
	1	Input	228
	2	Input	57
intestine LIN-35	1*	GFP	569
	2	GFP	92
	1	Input	344
	2	Input	141
intestine HPL-2	1*	GFP	513
	2	GFP	160
	1	Input	275
	2	Input	69

Asterisk denotes the replicate used in figures showing binding profiles.

Supplemental materials and methods

Genetics of *efl-1* mutants

The *efl-1* locus exists in a region of the genome that does is not covered by a balancer chromosome. The *efl-1(n3318)* allele that exhibits the phenotype of interest is in a strain (MT9926) in which the chromosome carrying the wild type *efl-1* allele is marked by a double mutation instead. One cannot distinguish *efl-1* homozygotes from heterozygotes until late adulthood, making the identification of whether the sterile phenotype is rescued late, limited to a subset of progeny, and reliant on a somewhat tenuous statistical argument. Given the extensive similarity of EFL-1 binding sites in the soma of L1 larvae regardless of whether the native protein or the transgene was assessed, we believe that the transgenic protein is mirroring the binding capability of the endogenous protein. Moreover, the extensive overlap between EFL-1 and DPL-1 binding sites in all tissues, coupled with the rescue of *dpl-1* mutant phenotypes by transgenic DPL-1 in both the soma and germ line also argues that these proteins are functional.

Selection criteria to define tissue-specific sets of binding sites and targets

Germline-specific sites. Previous studies indicated that *lin-35* mutants do not have the same germline phenotype as *efl-1* or *dpl-1* mutants, and that LIN-35 appeared to repress gene expression in the germline through an E2F-independent mechanism, while EFL-1/DPL-1 activate genes involved in oogenesis, ovulation and fertilization (Chi and Reinke, 2006). Thus, LIN-35 appears to act differently from EFL-1 and DPL-1 in the germ line. Additionally, from browsing the ChIP-seq tracks and examining the overlaps between factors, we observed that EFL-1 and DPL-1 bound very similar peaks in the germ line, but that LIN-35 bound many fewer (though mostly overlapping) sites. Therefore, we required that both EFL-1 and DPL-1 bound to the same peak (with a >10bp overlap) to call it a “germline” peak, but did not require LIN-35

binding to be significant. To exclude peaks that were also bound in other tissues, we removed peaks that overlapped with endogenous DPL-1 peaks, because this was one of the most robust somatic datasets that we had generated. The “germline” peaks that remained after removal of those bound by endogenous DPL-1 were visually examined. The vast majority were bound almost exclusively in the germ line, indicating that this exclusion criteria was sufficient to make a “germline-specific” set of binding sites.

Soma-specific sites. Previous studies had indicated that *lin-35*, *efl-1* and *dpl-1* act together to regulate diverse processes including vulval development as part of the SynMuvB pathway (reviewed in (Fay and Yochem, 2007). We therefore required that a peak had to be bound by all three factors expressed under their endogenous promoters in L1 animals (and therefore essentially only somatic tissues) in order to consider a peak truly a “soma” peak. To exclude peaks that were also bound in the germ line, we removed any peaks bound by germline EFL-1, which exhibited the most robust binding in the germ line. Visual assessment indicated that this criteria was quite effective. Because the intestine is one of the somatic tissues in L1 animals, the binding sites for factors expressed under their endogenous promoters were (almost always) present in the intestine as well, so we did not systematically exclude peaks bound by intestine LIN-35, intestine EFL-1, or intestine DPL-1. However, we noted that a subset of somatic peaks were poorly bound by intestine HPL-2, and that these appeared to correspond to less "HOT" sites. We therefore excluded peaks bound by intestine HPL-2 to define our most stringent set of soma-specific sites.

Intestine-specific sites. Based on visual examination, binding peaks by intestine LIN-35 and intestine HPL-2 fell into two general categories: binding in discrete, relatively narrow “typical” peaks close to gene start sites that overlapped considerably with soma- or broadly-bound peaks by DPL-1 and EFL-1, and broad binding peaks that correlated relatively poorly with gene starts, and were almost exclusively bound by intestine HPL-2 and intestine LIN-35. To define these intriguing broad binding peaks, we selected peaks bound by both intestine LIN-

35 and intestine HPL-2, but excluded intestine EFL-1 peaks and somatic EFL-1 peaks. Exclusion of these two sets of EFL-1 peaks was effective at enriching for the broad intestine-specific LIN-35/HPL-2 peaks. We did not exclude intestine or soma DPL-1 because we noted that sometimes weak DPL-1 could be seen at these broad sites. In addition to the broad peaks, a subset of discrete peaks at gene starts that are specific to intestine LIN-35 and HPL-2, are present in this set.

Broadly-bound sites. To define binding sites that are bound in both the germ line and at least one somatic tissue (the intestine), if not more, we required binding by the most robust factor in each tissue: germline EFL-1, intestine DPL-1, and somatic LIN-35. This criteria appeared to be sufficient to define most binding sites that showed consistently high binding across all tissues.

Venn diagram bioinformatics

To compare binding sites between factors and tissues, the binding site intervals for each factor in each tissue were loaded into the Galaxy toolbase and overlapping intervals were determined in pair-wise comparisons between any two factors or tissues using the "intersect genomic intervals" option, with an acceptable overlap of 10 bp between any two intervals. The overlaps were depicted in Venn diagrams that were hand-drawn to approximate the relative levels of overlap and are not meant to be precise.

ChIP-qPCR

We used ChIP material collected as described in methods on separate biological replicates that underwent a separate IP from those used for ChIP-seq. Reactions were carried out in duplicate for each primer set in a 25 µl volume using iQ SYBR Green Supermix (Bio-Rad, Hercules, CA). The PCR reactions were performed on the Realplex 2 machine (Eppendorf, Hauppauge, NY). The PCR program was Step 1: 95ºC for 2 minutes; Step 2: 95ºC for 15 seconds; Step 3: 55ºC for 15 seconds; Step 4: 68ºC for 20 seconds. Steps 2-4 were repeated

40 times; followed by a standard melting curve. The enrichment value was calculated by subtracting the Ct value of a given gene in the GFP ChIP sample by the Ct value of the IgG sample after normalizing to the negative control *fer-1*.

MEME analysis

Consensus binding sites for each tissue were determined as follows. The binding site sequences from high confidence sites (within 500bp of start of at least one coding gene) were fetched using the Galaxy toolbase (http://main.g2.bx.psu.edu/). These sequences were then input into the web-based MEME-ChIP program (http://meme.sdsc.edu). This program selects the central 100-bp of the binding site sequence for analysis by MEME (Bailey and Elkan, 1994). In the case of the broadly-bound dataset, 600 of the input 1773 sequences were selected at random by the program. Standard parameters provided by the program were used to return the top three significant sites from each tissue-specific dataset. The output for each dataset is provided in Additional file 8, and displays the individual sequences containing each motif. The statistical analysis performed by MEME to determine the E value is described at http://meme.sdsc.edu.
